# Molecular structure of 3-*O*-acetyl-11-keto-β-boswellic acid and its ʟ-alanine conjugate (AKBA-Ala) and their impact on lipoxygenases

**DOI:** 10.3762/bjoc.22.99

**Published:** 2026-09-03

**Authors:** Jan M Peschel, Paul M Jordan, Benjamin Friebe, Helmar Görls, Phil Köhler, Ulrich S Schubert, Oliver Werz, Michael Gottschaldt

**Affiliations:** 1 Laboratory of Organic and Macromolecular Chemistry (IOMC), Friedrich Schiller University Jena, Humboldtstraße 10, 07743 Jena, Germany; 2 Jena Center for Soft Matter (JCSM), Friedrich Schiller University Jena, Philosophenweg 7, 07743 Jena, Germany; 3 Department of Pharmaceutical/Medicinal Chemistry, Institute of Pharmacy, Friedrich Schiller University Jena, 07743 Jena, Germany; 4 Institute of Inorganic and Analytical Chemistry (IAAC), Friedrich Schiller University Jena, Humboldtstraße 8, 07743 Jena, Germany

**Keywords:** AKBA, amino acid functionalization, boswellic acids, 5-lipoxygenase, specialized pro-resolving mediators

## Abstract

3-*O*-Acetyl-11-keto-β-boswellic acid (AKBA) is an anti-inflammatory constituent of frankincense. Its potency is attributed to its ability to interact with enzymes in the inflammation cascade, such as lipoxygenase and cyclooxygenase, to decrease formation of pro-inflammatory products while increasing the production of pro-resolving mediators. However, the availability of AKBA in vivo may be limited by factors such as poor solubility and protein binding in serum. A prodrug approach, wherein AKBA is conjugated to amino acids through its carboxylate moiety, could be a suitable way to improve solubility and half-life in blood while maintaining anti-inflammatory efficacy. In this study, we describe a robust protocol for the preparation of high-purity amino acid-AKBA conjugates, exemplified by synthesis of the ʟ-alaninate derivative AKBA-Ala. The alanine conjugate was characterized by NMR spectroscopy, ESI-MS, HPLC, and X-ray crystallography, representing the first reported solid-state structure of an amino acid-AKBA conjugate. In comparison to AKBA, biological investigations of AKBA-Ala demonstrated a reduced inhibition (40% weaker at 10 µM) of 5-lipoxygenase (5-LOX) product formation in human neutrophils, but slightly enhanced production (120% higher at 10 µM) of specialized pro-resolving mediators (SPM) in human M2 macrophages. Overall, the anti-inflammatory potency of AKBA-Ala remains comparable to that of AKBA.

## Introduction

Frankincense (also known as olibanum) is the oleogum resin of several tree species in the *Boswellia* genus, mostly native to arid climates in northeastern Africa, southern Arabia, and central India [1]. It has held great cultural significance since antiquity, from its use as incense in religious ceremonies to perfumes and various applications in traditional medicine [2]. Clinical trials of frankincense extracts have suggested promising efficacy in the treatment of inflammatory diseases such as asthma, rheumatoid arthritis, osteoarthritis, Crohn’s disease, and collagenous colitis [3–5]. These favorable properties of the complex mixtures have prompted detailed investigation of the anti-inflammatory effects of the individual components and are largely attributed to their boswellic acid content [6–7]. Boswellic acids are a group of bioactive pentacyclic triterpenes found in *Boswellia* resin. The resin contains approximately 30% organic acids, and more than half of this fraction consists of boswellic acids, though the exact composition varies depending on species [8].

The most prominent boswellic acid derivative is 3-*O*-acetyl-11-keto-β-boswellic acid (AKBA), which interferes with several enzymes involved in the production of lipid mediators (LMs) that regulate the inflammatory response [4]. AKBA was identified as a potent inhibitor of arachidonate 5-lipoxygenase (5-LOX) [9] as well as cyclooxygenase-1 (COX-1) [10], which convert arachidonic acid (AA) into pro-inflammatory leukotrienes (LT) and prostaglandins (PG), respectively. Recent findings indicate a more complex interaction with LOXs, in which AKBA acts as an allosteric modulator of 5-LOX, promoting the production of specialized pro-resolving mediators (SPMs) over LTs by altering the regiospecificity of the enzyme [11–12]. AKBA also acts as an allosteric activator of 15-LOX-1, which contributes to the biosynthesis of SPM [13]. SPMs include a range of LMs derived from polyunsaturated fatty acids (PUFA, namely resolvins (Rv), protectins, and maresins) and lipoxins (LX), which contribute to the resolution of inflammation and tissue regeneration through different receptor-mediated pathways [14–16]. AKBA has also been found to block the nuclear factor kappa beta (NF-κB), resulting in reduced expression of associated pro-inflammatory, anti-apoptotic, proliferative, and angiogenic gene products [17–18]. Tumor-promoting inflammation has been dubbed one of the essential hallmarks of cancer [19]. Consequently, the anti-inflammatory properties of AKBA, in particular the NF-κB inhibition, render it a promising candidate for cancer therapy, with numerous studies demonstrating antiproliferative and pro-apoptotic effects, particularly in colorectal and prostate cancers [20–21].

While AKBA strongly inhibits 5-LOX in various cells, the inhibitory effect is greatly diminished in the presence of whole blood or serum due to albumin binding [9]. Moreover, AKBA has a poor bioavailability due to low solubility, unfavorable absorption, and short half-life after oral intake [4]. To overcome barriers in drug delivery, a prodrug approach could be a simple solution that does not require sophisticated drug delivery systems. Conjugation of AKBA, e.g., to peptides or hydrophobic residues via click chemistry has been found to alter solubility, influence biodistribution, and modulate anti-inflammatory activity [22–23]. Notably, it has been demonstrated that peptide-AKBA conjugates with PepH3 and Angiopep2 connected through a cleavable ester are capable of brain-targeted drug delivery and release after intravenous administration, without the need for additional nanocarriers [23–24]. These findings make peptide-AKBA conjugates particularly interesting for targeted drug delivery. In comparison to conjugation via an ester linker, the direct functionalization of AKBA’s carboxylate group with amino acids may increase its half-life in blood where amides are typically stable, and facilitate its release in protease-rich environments, such as macrophage lysosomes, tumor tissue, liver, or colon [25–27]. While the direct conjugation of AKBA to amino acids has been previously claimed by Naeini et al. [28], to us the synthetic procedure proposed therein appears to be inadequate for the synthesis and isolation of pure amino acid-AKBA conjugates. The analytical data provided suggest that little to no conjugates were formed, and no efforts were undertaken to purify the products.

Herein, we report a method for the preparation of high purity amino acid-AKBA conjugates exemplified by the synthesis of the ʟ-alanine conjugate AKBA-Ala. The acquired conjugate was characterized by ESI-MS, NMR spectroscopy, and HPLC. Additionally, the solid-state molecular structures of AKBA and its alanine conjugate were determined by X-ray crystallography. While the molecular structure of AKBA has been previously reported (including a reported structure of a mixed crystal of AKBA and brominated AKBA) [29–30], to the best of our knowledge this constitutes the first reported solid-state molecular structure of any amino acid-AKBA conjugate. Finally, the capacity of AKBA-Ala to inhibit 5-LOX product formation in human neutrophils and to induce SPM biosynthesis in primary human M2 macrophages was evaluated in comparison to AKBA.

## Results and Discussion

### Synthesis of AKBA-Ala

**Scheme 1:**
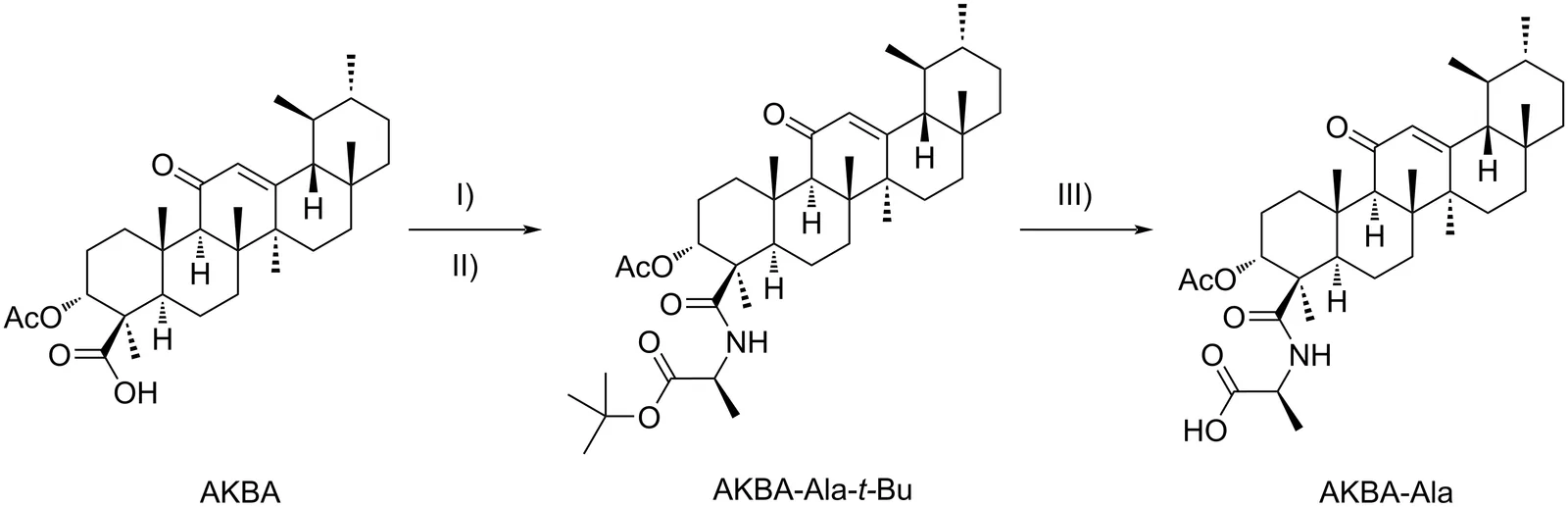
Schematic representation of the synthesis of AKBA-Ala. I) SOCl_2_, toluene, 20 °C, 18 h. II) ʟ-Alanine *tert*-butyl ester hydrochloride, 4-(dimethylamino)pyridine, NEt_3_, CH_2_Cl_2_, 0 °C → 20 °C, 72 h. III) Trifluoroacetic acid, CH_2_Cl_2_, 0 °C → 20 °C, 24 h.

The synthetic route to AKBA-Ala is displayed in Scheme 1. To introduce the amino acid, we chose commercially available *tert*-butyl-protected ʟ-alanine hydrochloride. Protection of the carboxylate group minimizes side reactions in the amide coupling step and the *tert*-butyl group can be readily removed by trifluoroacetic acid under anhydrous conditions without risking hydrolysis of the C3α-acetate group.

**Figure 1:**
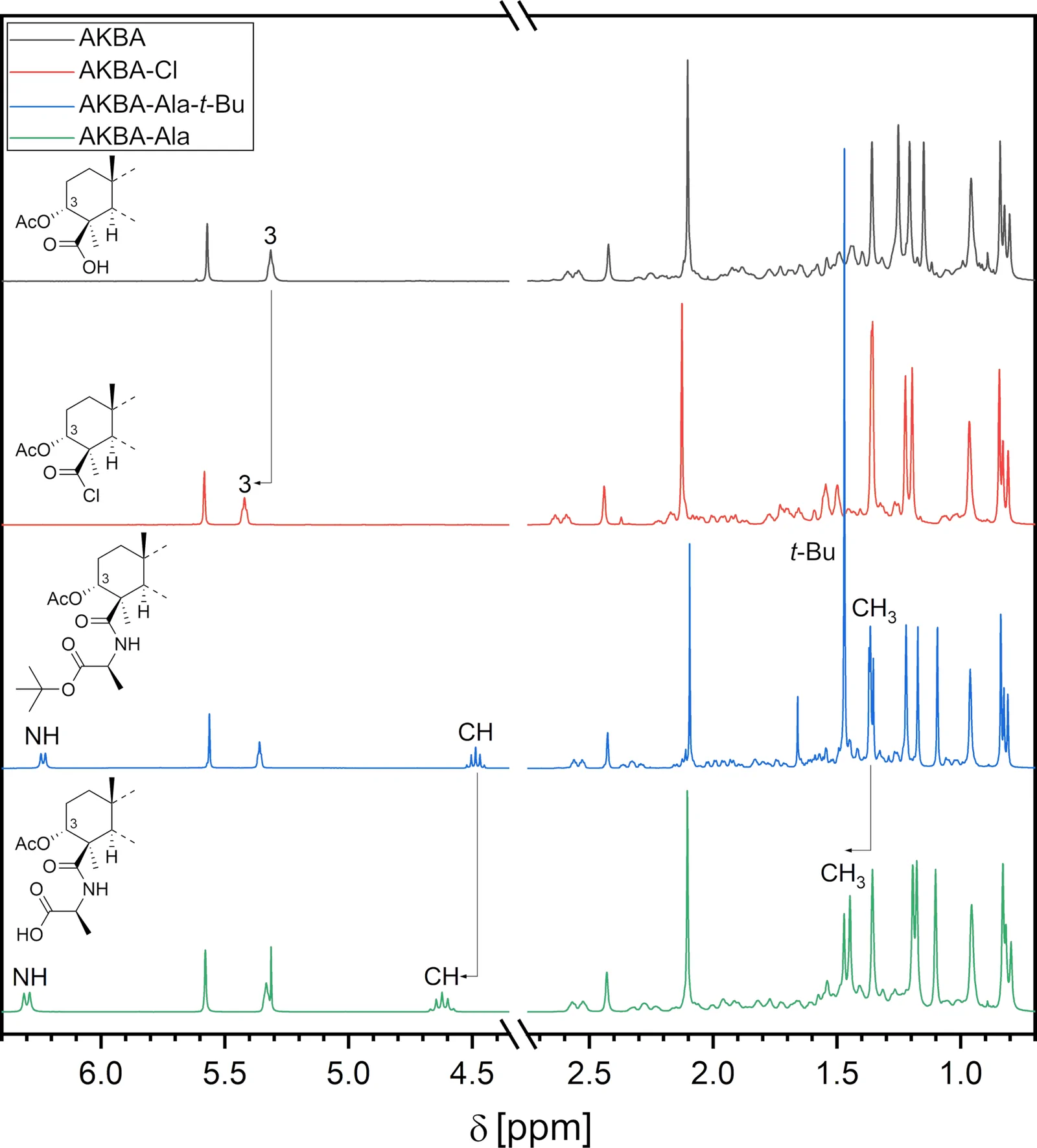
Stacked ^1^H NMR spectra of all products (AKBA and AKBA-Cl: 300 MHz; AKBA-Ala*-t-*Bu and AKBA-Ala: 400 MHz, CDCl_3_). For clarity, only a section of the molecules is displayed. For shift range 6.6 to 15 ppm see Supporting Information File 1, Figure S1.

For the amide coupling step, AKBA was first converted into its acid chloride by reaction with thionyl chloride in toluene. After removal of excess thionyl chloride and solvent, AKBA-Cl was obtained. The full conversion to the acid chloride was confirmed by an upfield shift of the carboxyl signal (C23: 182.15 ppm to 176.94 ppm) and a downfield shift of the nearby *ipso*-carbon signal (C4: 46.50 ppm to 56.91 ppm, Figure S7 in Supporting Information File 1). A downfield shift of signals belonging to nearby hydrogen atoms was also observed (Figure 1), and the carboxyl hydrogen signal disappeared (Supporting Information File 1, Figure S1). Due to air exposure, some hydrolysis occurred before the next reaction, as observed by ESI-MS and thin-layer chromatography (TLC).

AKBA-Cl was allowed to react with an excess (2 equiv) of *tert*-butyl-protected ʟ-alanine hydrochloride in the presence of triethylamine as the base and catalytic amounts of 4-(dimethylamino)pyridine. The acylation proceeds slowly at room temperature owing to the steric bulk of AKBA-Cl, but complete conversion of AKBA-Cl was accomplished after several days. After chromatography some amount of contamination was present in the product fraction, most likely AKBA due to its similar polarity (*R*_f_ 0.59 for AKBA and 0.57 for product in 1:1 *n*-pentane/diethyl ether with 1% acetic acid). As the deprotected AKBA-Ala is substantially more polar any leftover AKBA should be removable by column chromatography after the next step. The successful introduction of the *t-*Bu-Ala moiety was confirmed by the presence of corresponding signals in the ^1^H NMR spectrum (Figure 1) and ESI-MS (Supporting Information File 1, Figure S9). The ^13^C-APT-NMR spectrum likewise shows new signals belonging to *t-*Bu-Ala (Supporting Information File 1, Figure S10). One notable concern during the coupling of chiral amino acids such as ʟ-alanine is the possibility of racemization at the α-carbon. In the present case, the conditions for acid chloride coupling of *t-*Bu-Ala were unlikely to cause racemization of ʟ-alanine. Only mild organic bases were employed, the reaction temperature was kept low, and the carboxyl group of ʟ-alanine was not activated, thus avoiding the formation of more CH-acidic reactive intermediates such as oxazolones [31]. Furthermore, the ^1^H and ^13^C NMR spectra showed no additional signals that could be attributed to the alanine moiety beyond what is expected for a single diastereomer, suggesting that if any ᴅ-alanine conjugate was present, it was below the limit of detection for these techniques.

For the final step, AKBA-Ala*-t-*Bu was treated with 35% (v/v) trifluoroacetic acid in dry dichloromethane. The *tert*-butyl ester can be readily cleaved even in anhydrous conditions via an A_AL_1 mechanism, while the 3-*O*-acetate group of AKBA remains intact. NMR analysis confirmed the complete removal of the *t-*Bu group through the absence of the corresponding signals in the ^1^H and ^13^C NMR spectra (Figure 1 and Figure S13 in Supporting Information File 1). In the extended ^1^H NMR spectrum (Figure S1, Supporting Information File 1), the carboxylic acid signal appeared as a broad singlet at 9.0–7.5 ppm.

### X-ray crystallography of AKBA and AKBA-Ala

#### Molecular structure in the crystal

**Figure 2:**
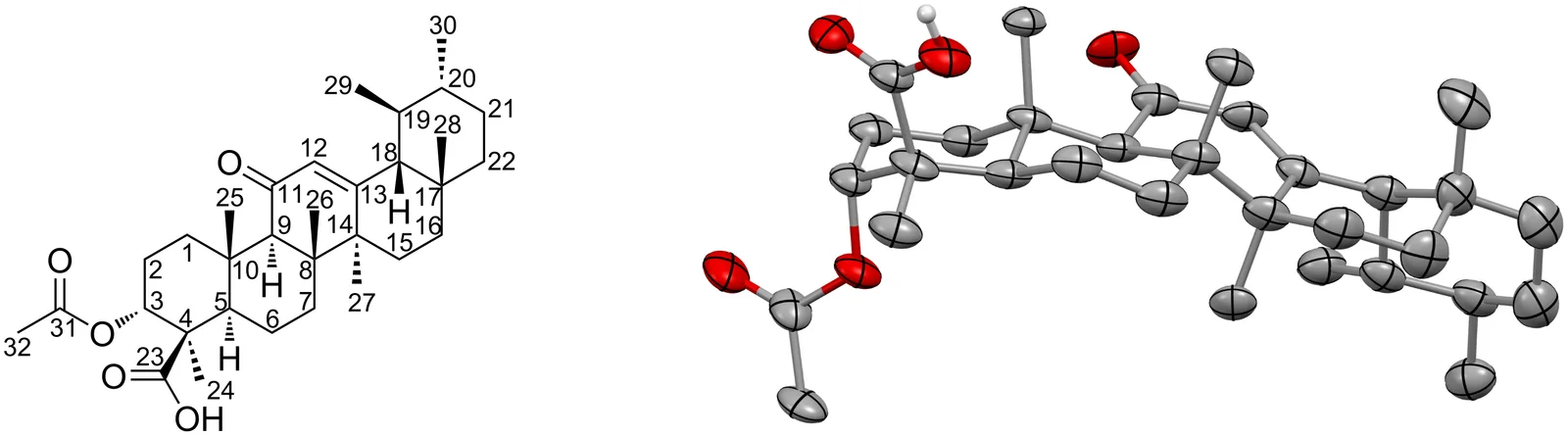
Displacement ellipsoid plot and atom-numbering scheme of AKBA (drawn at 30% probability; one of two molecules in the asymmetric unit). Hydrogen atoms attached to C-atoms are omitted for clarity. Colors: C (grey), H (white), O (red).

**Figure 3:**
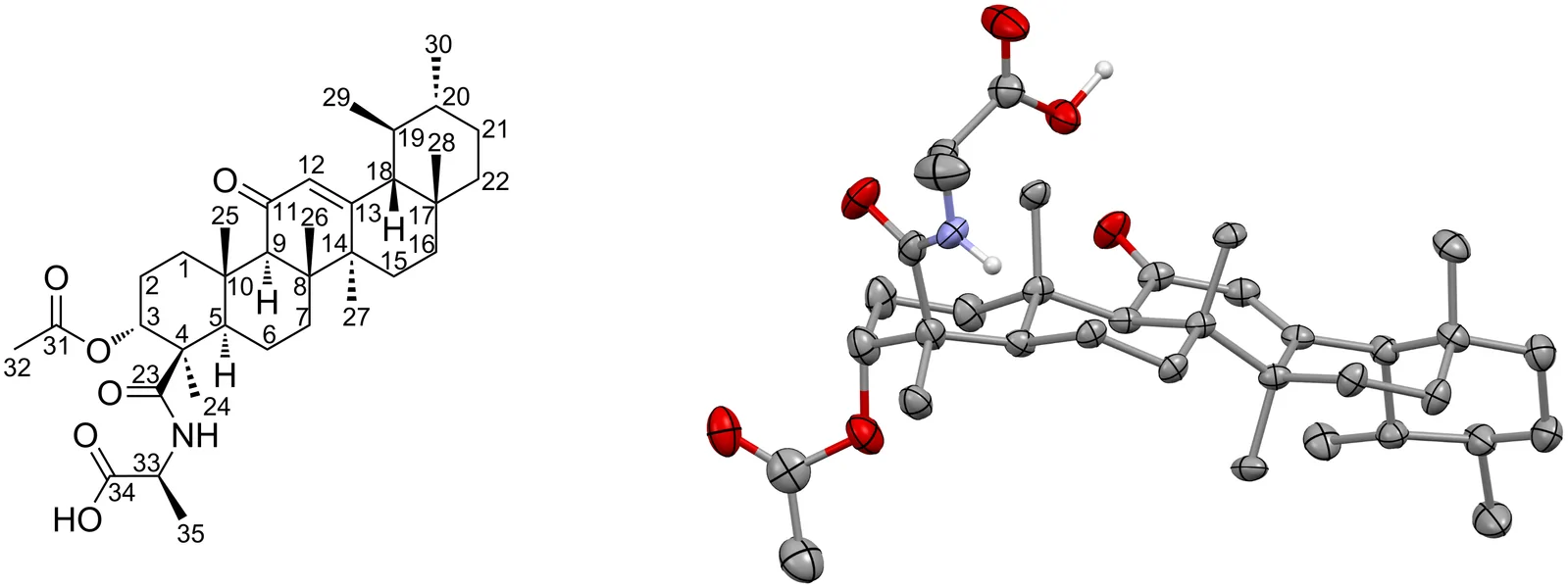
Displacement ellipsoid plot and atom-numbering scheme of AKBA-Ala (drawn at 50% probability). Hydrogen atoms attached to C-atoms are omitted for clarity. Colors: C (grey), H (white), O (red), and N (blue).

The solid-state molecular structures of AKBA and AKBA-Ala are displayed in Figure 2 and Figure 3, the unit cell measurements of both compounds are compared in Supporting Information File 1, Table S1.

The configuration of AKBA matches literature results [29–30]. Briefly, the four cyclohexane rings containing C1–C18 feature an all equatorial arrangement with only the C17–C22 ring in axial position. The α,β-unsaturated ketone enforces a nearly planar arrangement of C9 and C11–14 in the third ring, all other rings feature chair conformation. The 4-carboxyl group and the 3-acetoxy group are both in axial position sticking out from the sheet-like structure of the ring system. The AKBA molecules are arranged in linear chains connected by hydrogen bonds between the hydroxy group of the carboxylic acid and the C=O of the 3-*O*-acetate moiety (O–O-distance 2.692 Å, Supporting Information File 1, Figure S16). Adjacent molecules in the chain adopt alternating orientations related by an approximately 180° rotation.

The solid-state molecular structure of AKBA-Ala (Figure 3) shows the newly introduced ʟ-alanine moiety sticking out of the plane formed by the AKBA cyclohexane rings. Only minor changes in the conformation of AKBA were observed as a result of ʟ-alanine introduction. The increased steric bulk was reflected in a slightly increased distance between the axial C25 and C23 (3.149 Å in AKBA-Ala, 3.092 Å in AKBA), pushing the 4-amide group outward (O–C3–C4–C23 torsion angle 164.3° in AKBA, 163° in AKBA-Ala). The AKBA-Ala molecules form hydrogen-bonded zigzag chains with alternating orientations of adjacent molecules. Unlike in AKBA, hydrogen bonds of the COOH group of the ʟ-alanine moiety connect to the ketone at C11 rather than 3-*O*-acetate (O–O-distance 2.665 Å, Supporting Information File 1, Figure S17).

The wavelength (Mo-K_α_ = 0.71073 Å) did not allow distinction between AKBA and its enantiomer as no heavy atoms are present, so the absolute structure of AKBA could not be determined. In AKBA-Ala, because the configuration of the introduced alanine moiety was known to be the ʟ-isomer, it could act as a chiral auxiliary revealing the configuration of the attached AKBA to be as displayed in Figure 3.

#### Biological evaluations of anti-inflammatory properties of AKBA-Ala

**Figure 4:**
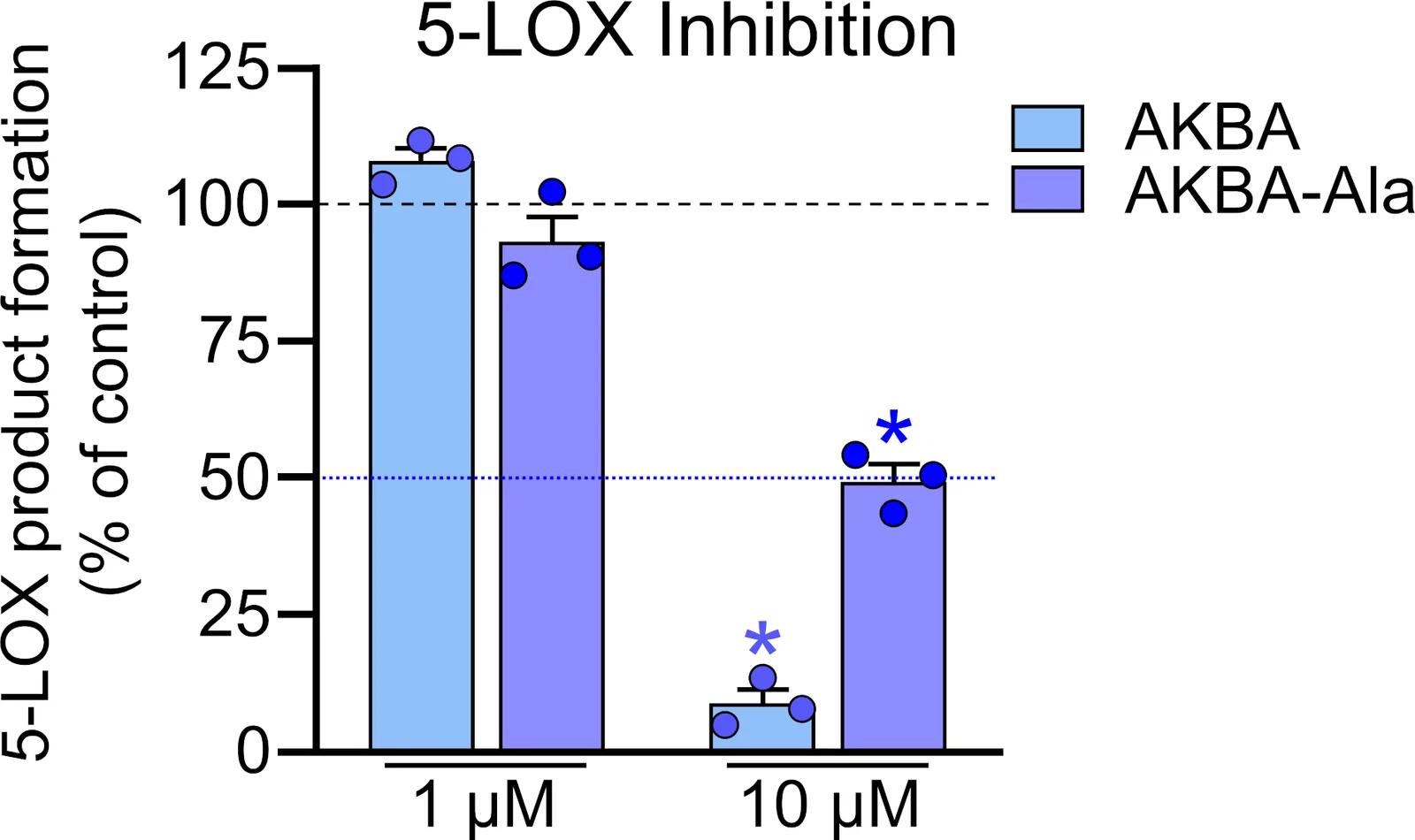
Inhibition of 5-LOX product formation by AKBA and AKBA-Ala in human neutrophils. Neutrophils were preincubated with AKBA or AKBA-Ala at 1 and 10 µM or control (0.1% DMSO) for 15 min at 37 °C and then stimulated with 2.5 µM A23187. After 10 min, the reaction was stopped, and 5-LOX products were extracted via solid-phase extraction (SPE) and analyzed with HPLC. Values are given as 5-LOX products (leukotriene B4 (LTB_4_), trans-LTB_4_, epi-trans-LTB_4_ and 5-hydroxyeicosatetraenoic acid (5-HETE)) in percentage of control. For statistical analysis a paired t-test versus vehicle was used, * < 0.05; *n* = 3 individual experiments.

AKBA exhibits anti-inflammatory properties by allosterically inhibiting 5-LOX product formation [12]. Therefore, we first assessed the potency of AKBA-Ala to inhibit 5-LOX product formation in Ca^2+^-ionophore A23187-stimulated human neutrophils in comparison with AKBA. At a concentration of 1 µM, neither AKBA-Ala nor AKBA affected 5-LOX product formation. However, at 10 µM, both compounds significantly reduced 5-LOX activity (Figure 4). Notably, AKBA demonstrated a stronger inhibitory effect, reducing 5-LOX product formation by approximately 85%, whereas AKBA-Ala inhibited 5-LOX activity by about 50% (Figure 4).

**Figure 5:**
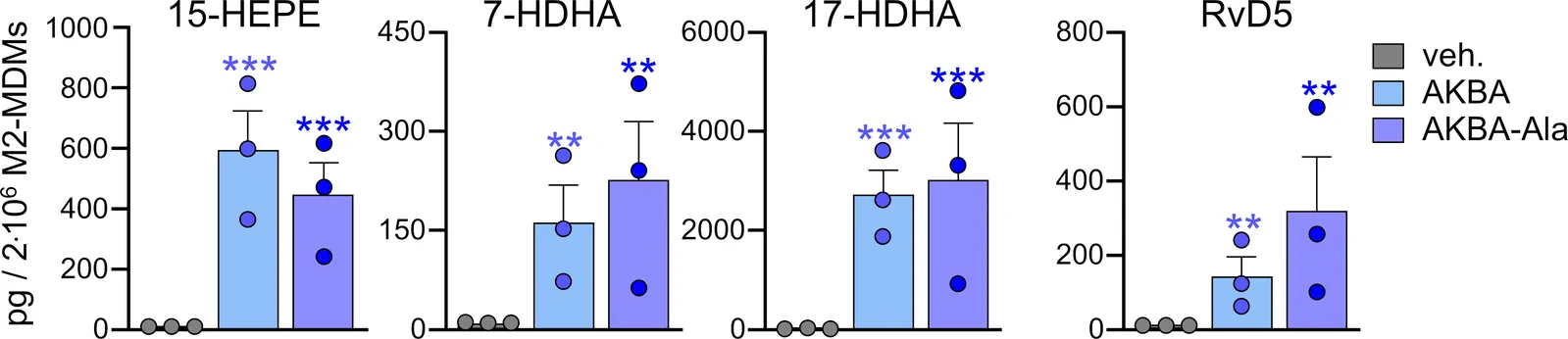
Induction of pro-resolving 15-LOX products and RvD5 by AKBA and AKBA-Ala in human M2-MDMs. M2-MDM (2 × 10^6^ cells) were incubated with 10 µM AKBA, 10 µM AKBA-Ala, or vehicle (0.1% DMSO) for 180 min at 37 °C. The formed LM were quantified in the supernatants using UPLC-MS/MS. 15-HEPE, 7-HDHA, 17-HDHA, and RvD5 are shown as pg/2 × 10^6^ M2-MDMs in mean ± S.E.M and in single values. Data were log-transformed for statistical analysis; one-way ANOVA with Dunnett's multiple comparisons test, **p < 0.01; ***p < 0.001 versus vehicle.

Besides the inhibition of pro-inflammatory LTs, AKBA induces the formation of 15-LOX-1 products and SPM in human primary M2 macrophages, leading to a faster resolution of the inflammatory process [13]. We therefore investigated whether AKBA-Ala similarly stimulates pro-resolving LM formation. At a concentration of 10 µM, AKBA-Ala increased the formation of eicosapentaenoic acid (EPA)-derived 15-HEPE (15-hydroxyeicosapentaenoic acid) as well as docosahexaenoic acid (DHA)-derived 7-HDHA (7-hydroxydocosahexaenoic acid) and 17-HDHA (17-hydroxydocosahexaenoic acid) in human M2-MDM (monocyte-derived macrophages) to a comparable extent as AKBA (Figure 5). Interestingly, AKBA-Ala induced slightly higher levels of the SPM resolvin D5 (RvD5) compared with AKBA (Figure 5). Overall, these findings demonstrate that the anti-inflammatory biological potency of AKBA-Ala is comparable to that of AKBA, with only minor differences.

## Conclusion

AKBA was functionalized with an ʟ-alanine moiety at the carboxylate group through a series of three simple reactions. The successful synthesis and isolation of AKBA-Ala was verified by NMR spectroscopy, ESI-MS, and HPLC. Furthermore, the solid-state molecular structures of AKBA and AKBA-Ala were determined by X-ray crystallography. The use of a *tert*-butyl protecting group at the carboxyl group of the amino acid enables clean conjugation and subsequent deprotection by TFA while leaving the 3-*O*-acetyl group of AKBA intact. In direct comparison to AKBA, the conjugate AKBA-Ala revealed slightly decreased potency in human neutrophils to inhibit 5-LOX, but in human M2-MDM it increased the formation of inflammation-resolving LM, suggesting a strong anti-inflammatory potential.

This protocol should be applicable for the conjugation of any amino acid (or amino-acid sequence, including proteins) as long as its side chain tolerates the presence of acyl chloride. For amino acids with protic side chains (e.g., serine, lysine or cysteine) additional TFA-cleavable protecting groups at the side chain would be necessary, similar to what is universally applied in Fmoc-based solid-phase peptide synthesis [32].

## Supporting Information

Supporting information features a list of abbreviations, detailed experimental procedures, ^1^H and ^13^C NMR spectra, ESI mass spectra, and HPLC data. Crystallographic data (excluding structure factors) has been deposited with the Cambridge Crystallographic Data Centre as supplementary publication CCDC-2517232 for AKBA, and CCDC-2517233 for AKBA-Ala. Copies of the data can be obtained free of charge on application to CCDC, 12 Union Road, Cambridge CB2 1EZ, UK [Email: deposit@ccdc.cam.ac.uk].

File 1 Experimental part and analytical data.

## Data Availability

Data generated and analyzed during this study are available from the corresponding author upon reasonable request.
