# Supplementary material for: Molecular structure of 3-O-acetyl-11-keto-β-boswellic acid and its ʟ-alanine conjugate (AKBA-Ala) and their impact on lipoxygenases
Source: Beilstein J Org Chem. 2026 Sep 3;22:1229–36. doi: 10.3762/bjoc.22.99 (PMC13575334; doi:10.3762/bjoc.22.99)
Supplement: Supplementary file 1 [file Beilstein_J_Org_Chem-22-1229-s001.pdf]

BEILSTEIN JOURNAL OF ORGANIC CHEMISTRY

Supporting Information

for

Molecular structure of 3-*O*-acetyl-11-keto-β-boswellic acid and its L-alanine conjugate (AKBA-Ala) and their impact on lipoxygenases

Jan M. Peschel, Paul M. Jordan, Benjamin Friebe, Helmar Görls, Phil Köhler,
Ulrich S. Schubert, Oliver Werz and Michael Gottschaldt

Beilstein J. Org. Chem. **2026,** *22,* 1229–1236. doi:10.3762/bjoc.22.99

Experimental part and analytical data

License and Terms: This is a supporting information file under the terms of the Creative Commons Attribution License (https://creativecommons.org/licenses/by/4.0). Please note that the reuse, redistribution and reproduction in particular requires that the author(s) and source are credited and that individual graphics may be subject to special legal provisions.
The license is subject to the *Beilstein Journal of Organic Chemistry* terms and conditions: (https://www.beilstein-journals.org/bjoc/terms)

Abbreviations

AA: arachidonic acid

Ala: L-alanine

AKBA: 3-*O*-acetyl-11-keto-β-boswellic acid

APT: attached proton test

ESI-MS: electrospray ionization mass spectrometry

HPLC: high-pressure liquid chromatography

IL-4: interleukin 4

LM: lipid mediator

LT: leukotriene

LX: lipoxin

LOX: lipoxygenase

M-CSF: macrophage colony stimulating factor

NMR: nuclear magnetic resonance

PG: prostaglandin

TLC: thin-layer chromatography

1 Experimental data

1.1 Materials

Materials for synthesis

Frankincense (the gum resin derived from *Boswellia sacra*) was purchased from Willy Benecke, Hamburg, Germany. L-alanine *tert*-butyl ester hydrochloride (≥ 98%) and trifluoroacetic acid (TFA, ≥99%) were purchased from TCI. Acetic acid (AcOH, ≥99.7%) and hydrochloric acid (HCl, 37%) were purchased from Fisher Scientific. Acetic anhydride (≥99%) was purchased from Grüssing, Pyridine (≥99.8%), 4-(dimethylamino)pyridine (4-DMAP, ≥99%), *N*-bromosuccinimide (NBS, ≥98%), thionyl chloride (≥99%) and dry triethylamine (NEt_3, ≥99.5%) were purchased from Sigma Aldrich. Acetonitrile (≥99.9%), dichloromethane (CH_2Cl_2, ≥99.8%), diethyl ether (≥99.5%), 1,4-dioxane (≥99.5%), ethyl acetate (≥99%), *n*-pentane (≥99%), *n*-heptane (≥99%), barium hydroxide octahydrate (≥98%), calcium carbonate (≥98.5%), magnesium sulfate (≥99%), sodium sulfate (≥98.5%) and sodium bicarbonate (≥99.5%) were purchased from Carl Roth. Dry solvents toluene (≥99.8%) and dichloromethane (≥99.9%) were received from Acros Organics. Reaction progress was monitored by thin-layer chromatography (TLC) on silica gel 60 F_{254} coated aluminum plates (Sigma Aldrich) using UV light at 254 nm and potassium permanganate stain [1]. Column chromatography was performed on a glass column with silica gel 60 (Fluka). Solvents used in chromatography were distilled before use. All other chemicals were used without further purification.

Materials for biological investigations

Cell culture media as well as supplemental reagents were purchased from Sigma Aldrich unless stated otherwise. Cell culture flasks were purchased from Greiner Bio-One. Ca^{2+}-ionophore A23187 and lipid mediator (LM) standards were purchased from Cayman Chemical.

1.2 Instrumentation

Photooxidation was carried out in a photoLAB B400-700 BASIC BATCH-L (Peschl Ultraviolet) photoreactor using a novaLIGHT TQ150 medium pressure mercury lamp (150 W, 200–600 nm). HPLC was performed on a Chromolith High Resolution RP-18ec 100 × 4.6 (CH_3CN/0.1% H_3PO_4 90:10, 35 °C; 1.5 mL/min, UV detection at 250 nm). 1D-NMR spectra were recorded at room temperature on one of the following devices: Bruker Avance I (300.13 MHz, ^{1}H; 75.5 MHz, ^{13}C) with a 5 mm BBF-1H/D z-Grad probehead and B-ACS 120 sample changer; Bruker Avance II (400.13 MHz, ^{1}H; 100.62 MHz, ^{13}C) with a 5 mm PA BBF-H-D-05 Z probehead and analysis-ACS 60 sample changer. In addition, a Bruker Avance III 600 (600.15 MHz, ^{1}H; 150.9 MHz, ^{13}C) with a 5 mm QCI CP2 cryoprobe and automatic sample loading system (SampleCase) for high-throughput sample analysis was used to measure 2D-NMR spectra for signal assignment. Chemical shifts (δ) are expressed in parts per million (ppm) relative to tetramethyl silane. NMR spectra were processed using ACD Spectrus Processor 2023.2.4 or Bruker TopSpin 4.4.1 by applying phase correction, baseline correction, and matching the chemical shift of the solvent peak to a reference value as necessary. Electrospray ionization mass spectrometry (ESI-MS) was performed on a Bruker MicrOTOF-QII mass spectrometer.

For structure determination, the intensity data for the compounds were collected on a Bruker-Nonius KappaCCD diffractometer equipped with a Mo-Kα IμS microfocus source and an Apex2 CCD detector. Data were corrected for Lorentz and polarization effects; absorption was considered on a semi-empirical basis using multiple scans. [2-5] The crystal structures were solved with SHELXT-2018/3 [6] and refined by full matrix least-squares methods on F^2 with SHELXL-2018/3 [7], using the Olex 1.2 environment [8]. The hydrogen atoms bonded to the hydroxy-group O5 and the amine-group N1 of AKBA-Ala were located by difference Fourier synthesis and refined isotropically. All other

hydrogen atoms were included at calculated positions with fixed thermal parameters. All non-hydrogen atoms were refined anisotropically [7,8].

1.3 Methods

1.3.1 Synthesis

Preparation of AKBA from frankincense

3-Acetyl-11-keto-β-boswellic acid (AKBA) was obtained according to a modified procedure published in reference [9]. 500 g of frankincense were extracted continuously with 1.5 L diethyl ether until completion. The extract was emulsified in 5 L of saturated aqueous barium hydroxide solution. The precipitated boswellic acids were filtered off and dissolved in a mixture of diethyl ether and 6 M hydrochloric acid (5 L each). The organic phase was dried with sodium sulfate, filtered, and volatiles were removed under reduced pressure. 46.2 g of the boswellic acids were dissolved in 185 mL of dry toluene. 29.6 mL (0.37 mol) of dry pyridine, 23.1 mL (0.24 mol) of acetic anhydride and 5.54 g (45 mmol) of 4-(dimethylamino)pyridine were added as a solution in 75 mL of dry toluene. Subsequent to refluxing for 3 h, 600 mL of cold 1 M hydrochloric acid were added. The organic phase was separated, and the aqueous phase was extracted with toluene (3 × 100 mL). The combined organic phases were washed with brine, dried over sodium sulfate, and filtered. Removal of the solvents in vacuo yielded an orange foam (18.8 g), which was dissolved in 525 mL of peroxide-free dioxane. 134 mL of water, 14.1 g (0.14 mol) of calcium carbonate and 16.2 g (91 mmol) of *N*-bromosuccinimide were added. The mixture was stirred vigorously in a photoreactor under nitrogen atmosphere for 35 min to achieve the photooxidation. Filtration and removal of the volatiles under reduced pressure produced a brown foam. Column chromatography (silica, pentane/diethyl ether 4:1 (v/v) + 0.5 vol % acetic acid) yielded the raw product, which was further purified by recrystallization from methanol. Yield: 12.4 g. Purity by HPLC: 98.8%.

Single crystals suitable for X-ray crystallography were obtained by dissolving 628 mg AKBA in 15 mL 2:1 *n*-heptane/diethyl ether and allowing the solvent to slowly evaporate until AKBA

crystallized as colorless clusters of needles (see Figure S15 for crystallization, Table S1 for crystal data).

^{1}H NMR (300 MHz) and ^{13}C NMR (75 MHz) matched literature results [10].

ESI-MS: *m/z* calculated for $[C_{32}H_{48}O_5 + Na]^+$: 535.3394; found: 535.3390.

Synthesis of 3-*O*-acetyl-11-keto-β-boswellic acid chloride (AKBA-Cl)

AKBA (1040 mg) was dissolved in dry toluene (20 mL) under argon. Thionyl chloride (20 mL) was added, and the solution was stirred at room temperature overnight. Excess thionyl chloride and solvent were removed by vacuum distillation to yield AKBA-Cl as a white solid which was used in the next step without further purification.

^{1}H NMR (300 MHz, $CDCl_3$): δ [ppm] = 5.58 (s, 1H, 12), 5.42 (t, 1H, 3), 2.61 (m, 1H, 1), 2.44 (s, 1H, 9), 2.17 (m, 1H, 2), 2.12 (m, 1H, 16), 2.16 (s, 3H, 32), 2.02 (m, 1H, 6), 1.91 (m, 1H, 15), 1.74 (m, 1H, 6), 1.68 (m, 1H, 7), 1.68 (m, 1H, 2), 1.57 (d, 1H, 18), 1.52 (m, 1H, 7), 1.51 (m, 1H, 5), 1.50 (m, 1H, 22), 1.47 (m, 1H, 21), 1.40 (m, 1H, 19), 1.36 (s, 3H, 27), 1.35 (s, 3H, 24), 1.35 (m, 1H, 22), 1.32 (m, 1H, 21), 1.24 (m, 1H, 15), 1.23 (m, 1H, 1), 1.22 (s, 3H, 26), 1.19 (s, 3H, 25), 1.04 (m, 1H, 16), 0.96 (m, 1H, 20), 0.96 (s, 3H, 30) 0.83 (s, 3H, 28), 0.81 (s, 3H, 29).

^{13}C NMR (75 MHz, $CDCl_3$): δ [ppm] = 198.80 (11), 176.94 (23), 169.79 (31), 165.04 (13), 130.48 (12), 73.25 (3), 60.34 (9), 59.04 (18), 56.91 (4), 51.26 (5), 45.00 (8), 43.78 (14), 40.89 (22), 39.36 (19), 39.27 (20), 37.51 (10), 34.26 (1), 33.99 (17), 33.00 (7), 30.90 (21), 28.86 (28), 27.50 (16), 27.24 (15), 23.67 (24), 23.37 (2), 21.26 (32), 21.14 (30), 20.51 (27), 18.82 (6), 18.28 (26), 17.43 (29), 14.54 (25).

ESI-MS: *m/z* calculated for $[C_{32}H_{47}ClO_4+Na]^+$: 553.3034; found: 553.3055.

Synthesis of *tert*-butyl (3-*O*-acetyl-11-keto-β-boswelloyl)-L-alaninate (AKBA-Ala-*t*-Bu)

AKBA-Cl was dissolved in dry dichloromethane (40 mL) and cooled to 0 °C. L-Alanine *tert*-butyl ester hydrochloride (744 mg, 2 equiv), 4-(dimethylamino)pyridine (26 mg, 0.1 equiv) and triethylamine (1.674 mL, 6 equiv) were added, and the solution was stirred for 15 min at 0 °C. The mixture was then stirred for three days at room temperature until TLC (1% acetic acid in 1:1 *n*-pentane/diethyl ether) revealed nearly complete conversion of the starting material. The mixture was poured into water/brine 80:20 (mL/mL) and extracted with diethyl ether (3× 80 mL). The combined organic phases were dried over sodium sulfate and dried in vacuo to yield a beige foam (1510 mg). This was further purified by column chromatography (5:4 *n*-pentane/diethyl ether) to yield 1303 mg (= 1173 mg, 92% yield with purity ≈90% by NMR) of a white foam containing AKBA-Ala-*t*-Bu and some impurities.

^{1}H NMR (400 MHz, $CDCl_3$): δ [ppm] = 6.24 (d, 0.8H, NH), 5.57 (s, 1H, 12), 5.36 (t, 1H, 3), 4.49 (quin, 1H, 33), 2.55 (m, 1H, 1), 2.43 (s, 1H, 9), 2.33 (m, 1H, 2), 2.12 (m, 1H, 16), 2.11 (s, 3H, 32), 2.01 (m, 1H, 6), 1.92 (m, 1H, 15), 1.82 (m, 1H, 6), 1.75 (m, 1H, 7), 1.64 (m, 1H, 2), 1.58 (m, 1H, 7) 1.57 (d, 1H, 18), 1.51 (m, 1H, 22), 1.48 (s, 9H, 37), 1.48 (m, 1H, 21), 1.44 (m, 1H, 5), 1.42 (m, 1H, 19), 1.37 (d, 3H, 35), 1.37 (s, 3H, 27), 1.35 (m, 1H, 22), 1.32 (m, 1H, 21), 1.25 (m, 1H, 15), 1.23 (m, 1H, 1), 1.22 (s, 3H, 26), 1.18 (s, 3H, 24), 1.10 (s, 3H, 25), 1.04 (m, 1H, 16), 0.97 (m, 1H, 20), 0.97 (s, 3H, 30), 0.84 (s, 3H, 28), 0.83 (s, 3H, 29).

^{13}C NMR (100 MHz, $CDCl_3$): δ [ppm] = 199.12 (11), 174.72 (23), 172.63 (34), 170.14 (31), 164.69 (13), 130.64 (12), 82.16 (36), 73.70 (3), 60.44 (9), 59.05 (18), 50.43 (5), 48.73 (33), 46.59 (4), 45.11 (8), 43.78 (14), 40.95 (22), 39.38 (19), 39.32 (20), 37.55 (10), 35.00 (1), 34.01 (17), 33.16 (7), 30.95 (21), 28.88 (28), 27.97 (37), 27.58 (16), 27.26 (15), 24.63 (24), 23.88 (2), 21.40 (32), 21.17 (30), 20.56 (27), 19.13 (6), 18.84 (35), 18.17 (26), 17.46 (29), 13.16 (25).

ESI-MS: *m/z* calculated for $[C_{39}H_{61}NO_6+Na]^+$: 662.4367; found: 662.4391.

Synthesis of (3-*O*-acetyl-11-keto-β-boswelloyl)-L-alanine (AKBA-Ala)

AKBA-Ala-*t*-Bu (1286 mg) was dissolved in dry dichloromethane (10 mL) and cooled to 0 °C. Trifluoroacetic acid (5.5 mL) was added slowly, and the solution was stirred for 1 h at 0 °C before being stirred overnight while warming to room temperature. After 24 h, TLC confirmed full conversion of the starting material (3:1 *n*-heptane/ethyl acetate). Dichloromethane (30 mL) was added, and the mixture was neutralized by addition of saturated sodium bicarbonate solution (70 mL), leading to the formation of a cloudy emulsion. The organic layer was separated by acidification with hydrochloric acid (pH 4 to 5) followed by centrifugation (8000 rpm, 9 min). The aqueous layer was removed with a syringe, and the organic phase was dried over sodium sulfate and concentrated in vacuo to yield a beige solid (1066 mg). The product was purified by column chromatography in two steps (eluent 1:1 *n*-heptane/ethyl acetate until elution of side product, then addition of 2% acetic acid to elute the product) to yield 838 mg (71% over 3 steps) of AKBA-Ala as a white powder. Purity by HPLC: 96.8%.

Single crystals suitable for X-ray crystallography were obtained by dissolving 24 mg in 2.5 mL 3:2 dichloromethane/acetonitrile and slowly evaporating the solvent until AKBA-Ala crystallized as colorless needles (see Figure S15 for crystallization, Table S1 for crystal data).

^{1}H NMR (400 MHz, $CDCl_3$): δ [ppm] = 6.30 (d, 1H, NH), 5.58 (s, 1H, 12), 5.33 (t, 1H, 3), 4.62 (quin, 1H, 33), 2.55 (m, 1H, 1), 2.43 (s, 1H, 9), 2.27 (m, 1H, 2), 2.11 (m, 1H, 16), 2.10 (s, 3H, 32), 1.98 (m, 1H, 6), 1.91 (m, 1H, 15), 1.82 (m, 1H, 6), 1.73 (m, 1H, 7), 1.64 (m, 1H, 2), 1.56 (d, 1H, 18), 1.54 (m, 1H, 7), 1.51 (m, 1H, 22), 1.47 (m, 1H, 21), 1.45 (d, 3H, 35), 1.45 (m, 1H, 5), 1.42 (m, 1H, 19), 1.36 (m, 1H, 22), 1.36 (s, 3H, 27), 1.33 (m, 1H, 21), 1.23 (m, 1H, 1), 1.22 (m, 1H, 15), 1.20 (s, 3H, 26), 1.19 (s, 3H, 24), 1.10 (s, 3H, 25), 1.04 (m, 1H, 16), 0.96 (m, 1H, 20), 0.96 (s, 3H, 30), 0.83 (s, 3H, 28), 0.81 (s, 3H, 29).

^{13}C NMR (75 MHz, $CDCl_3$): δ [ppm] = 199.50 (11), 176.53 (23), 175.30 (34), 170.36 (31), 165.35 (13), 130.41 (12), 73.51 (3), 60.36 (9), 59.04 (18), 50.46 (5), 48.10 (33), 46.61 (4), 45.09 (8), 43.78 (14), 40.89 (22), 39.39 (19), 39.26 (20), 37.48 (10), 34.84 (1), 33.98 (17), 33.14 (7), 30.90 (21), 28.88 (28), 27.50 (16), 27.22 (15), 24.62 (24), 23.82 (2), 21.36 (32), 21.17 (30), 20.47 (27), 19.08 (6), 18.19 (35), 18.18 (26), 17.41 (29), 13.30 (25).

ESI-MS: *m/z* calculated for $[C_{35}H_{53}NO_6+Na]^+$: 606.3758; found: 606.3765.

1.3.2 Isolation of neutrophils and generation of monocyte-derived macrophages (MDM)

Leukocyte concentrates, acquired from freshly withdrawn blood from healthy adult male and female volunteers (18 to 75 years old), were obtained from the Department of Transfusion Medicine at the University Hospital of Jena, Germany. The approval of the protocol was given by the ethical committee of the University Hospital Jena (approval no. 5050-01/17), and all methods were performed in accordance with the relevant guidelines and regulations. As previously described [11], leukocyte concentrates were mixed with dextran (MW 40,000, Leuconostoc) and the supernatants were centrifuged on a lymphocyte separation medium (Histopaque®-1077). Water-based hypotonic lysis eliminated remaining erythrocytes from the neutrophil fraction. After washing twice with ice-cold phosphate-buffered saline (PBS) at pH 7.4, the pelleted neutrophils were resuspended in 5 mL of PBS. After another washing step with ice-cold PBS, the peripheral blood mononuclear cell fraction (PBMC) was seeded in cell culture flasks in RPMI 1640, supplemented with 10% (v/v) heat-inactivated fetal calf serum (FCS), 100 U/mL penicillin and 100 µg/mL streptomycin, and kept for 1 h at 37 °C, 5% CO_2 for adherence. Monocytes were differentiated towards monocyte-derived macrophages (MDM) by exposure to M-CSF (20 ng/mL) for six days, and further polarized into M2-MDMs with IL-4 (20 ng/mL) for 48 h.

1.3.3 Determination of 5-LOX product formation in neutrophils

To assess the effects on 5-LOX product formation, human neutrophils were pre-incubated (5×10^6 in 1 mL) in PBS containing 0.1% glucose and 1 mM $CaCl_2$ with vehicle (DMSO, 0.1%), AKBA-Ala and AKBA at concentrations of 1 and 10 µM for 15 min at 37 °C. Ca^{2+}-ionophore A23187 at a concentration of 2.5 µM was used to stimulate the cells for 10 min at 37 °C. Following this, the incubation was stopped with 1 mL of ice-cold methanol containing 200 ng/mL prostaglandin B_1. 5-LOX products were isolated by solid-phase extraction and then separated and analyzed via RP-HPLC in accordance with an established protocol [12].

1.3.4 Induction of LM formation in human M2-MDMs and LM extraction

To examine whether AKBA-Ala and AKBA induce LM formation, M2-MDMs (2×10^6/mL) were incubated with vehicle (DMSO, 0.1%), AKBA-Ala and AKBA (at 10 µM) in PBS containing 1 mM $CaCl_2$ for 180 min at 37 °C. Afterwards, the reaction was stopped by adding 2 mL of ice-cold methanol containing deuterated LM standards (200 nM d_8-5S-HETE, d_4-LTB_4, d_5-LXA_4, d_5-RvD2, d_4-PGE_2 and 10 µM d_8-AA), and the samples were then processed for LM analysis using UPLC-MS/MS following a published protocol [13]. Samples were kept at −20 °C overnight to allow protein precipitation. Supernatants were collected after centrifugation (1200*g*, 10 min, 4 °C) and acidified by addition of 7 mL purified water pH 3.5 to each sample for solid-phase extraction (SPE). Before sample loading, C18 solid-phase cartridges (Waters) were washed with 6 mL methanol and equilibrated with 2 mL purified water. After washing with 6 mL H_2O and additional 6 mL *n*-hexane, LM were eluted with 6 mL of methyl formate. Finally, the samples were brought to dryness using an evaporation system (TurboVap LV, Biotage) and resuspended in 100 µL methanol/water 50:50 (v/v) for UPLC-MS/MS analysis.

1.3.5 UPLC-MS/MS analysis of LM

LMs and PUFAs were acquired via UPLC-MS/MS following an established protocol [13]. LMs were separated utilizing an Acquity™ UPLC system (Waters) equipped with an Acquity™ UPLC BEH C18 column (Waters; 1.7 µm, 2.1 × 100 mm) that was kept at 50 °C and the flow rate was set to 0.3 mL/min. Analytes were eluted using a gradient method with a mobile phase consisting of methanol, water, and acetic acid in a ratio of 42:58:0.01 (v/v/v) increasing up to 86:14:0.01 (v/v/v) over 12.5 min and finally set at 98:2:0.01 (v/v/v) for 3 min. LMs were detected using a QTRAP 5500 mass spectrometer (AB Sciex) equipped with electrospray ionization, operating in negative ionization mode using scheduled multiple reaction monitoring (MRM) combined with data-dependent acquisition. The MRM window was set to 60 s, optimized LM parameters (collision energy, entrance potential, declustering potential, collision cell exit potential) were adopted, and the curtain gas pressure was set at 35 psi. To confirm the retention time, external standards were used, and at least six diagnostic ions for each LM were used, as published previously [13]. The detected LMs were quantified via linear calibration curves, which were obtained for each analyte with r^2 values of 0.98 to 0.99, and the limit of quantification was determined.

Table S1: Crystal data and refinement details for the X-ray structure determinations.

Compound	AKBA	AKBA-Ala
formula	$C_{32}H_{48}O_5$	$C_{35}H_{53}NO_6$
formula weight ($g \cdot mol^{-1}$)	512.70	583.78
T/°*C*	–140(2)	–153(2)
crystal system	monoclinic	orthorhombic
space group	$P2_1$	$P2_12_12_1$
a/ Å	11.734(2)	8.120(1)
b/ Å	16.371(2)	16.121(2)
c/ Å	15.566(2)	24.276(3)
α/°	90	90
β/°	91.049(7)	90
γ/°	90	90
V/Å^3	2989.8(7)	3177.8(7)
Z	4	4
ρ ($g \cdot cm^{-3}$)	1.139	1.220
μ (cm^{-1})	0.75	0.82
measured data	31829	43939
data with $I > 2\sigma(I)$	6475	5550
unique data (R_{int})	10468/0.0742	6087/0.0504
wR_2 (all data, on F^2)[a]	0.3070	0.1669
R_1 ($I > 2\sigma(I)$) [a]	0.0986	0.0614
S [b]	1.014	1.052
Res. dens./$e \cdot Å^{-3}$	0.924/–0.268	0.711/–0.290
Flack parameter [14]	–0.1(8)	0.0(3)
absorpt. corr. method	multi-scan	multi-scan
absorpt. corr. $T_{min/max}$	0.5741/0.7456	0.9150/1.0000
CCDC No.	2517232	2517233

[a] Definition of the *R* indices: $R_1 = (\Sigma || F_o| - |F_c ||)/\Sigma |F_o|$;

$wR_2 = \{\Sigma[w(F_o^2-F_c^2)^2]/\Sigma[w(F_o^2)^2]\}^{1/2}$ with $w^{-1} = s^2(F_o^2) + (aP)^2+bP$; $P = [2F_c^2 + Max(F_O^2]/3$;

[b] $S = \{\Sigma[w(F_o^2-F_c^2)^2]/(N_o-N_p)\}^{1/2}$.

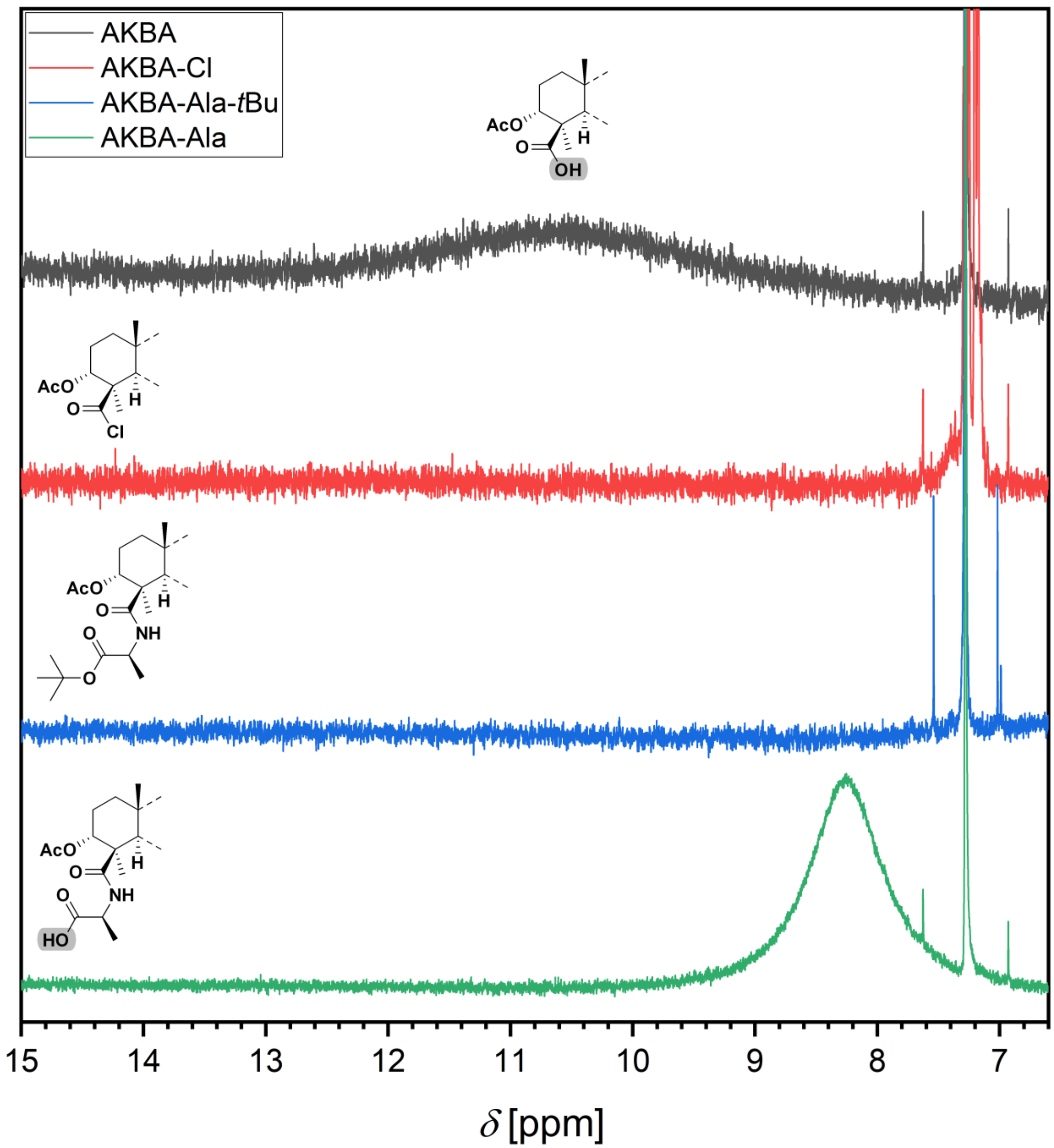

Figure S1: Extension of ^{1}H NMR spectra of all products (AKBA and AKBA-Cl: 300 MHz; AKBA-Ala-*t*-Bu and AKBA-Ala: 400 MHz, $CDCl_3$). For clarity, only a section of the molecules is displayed.

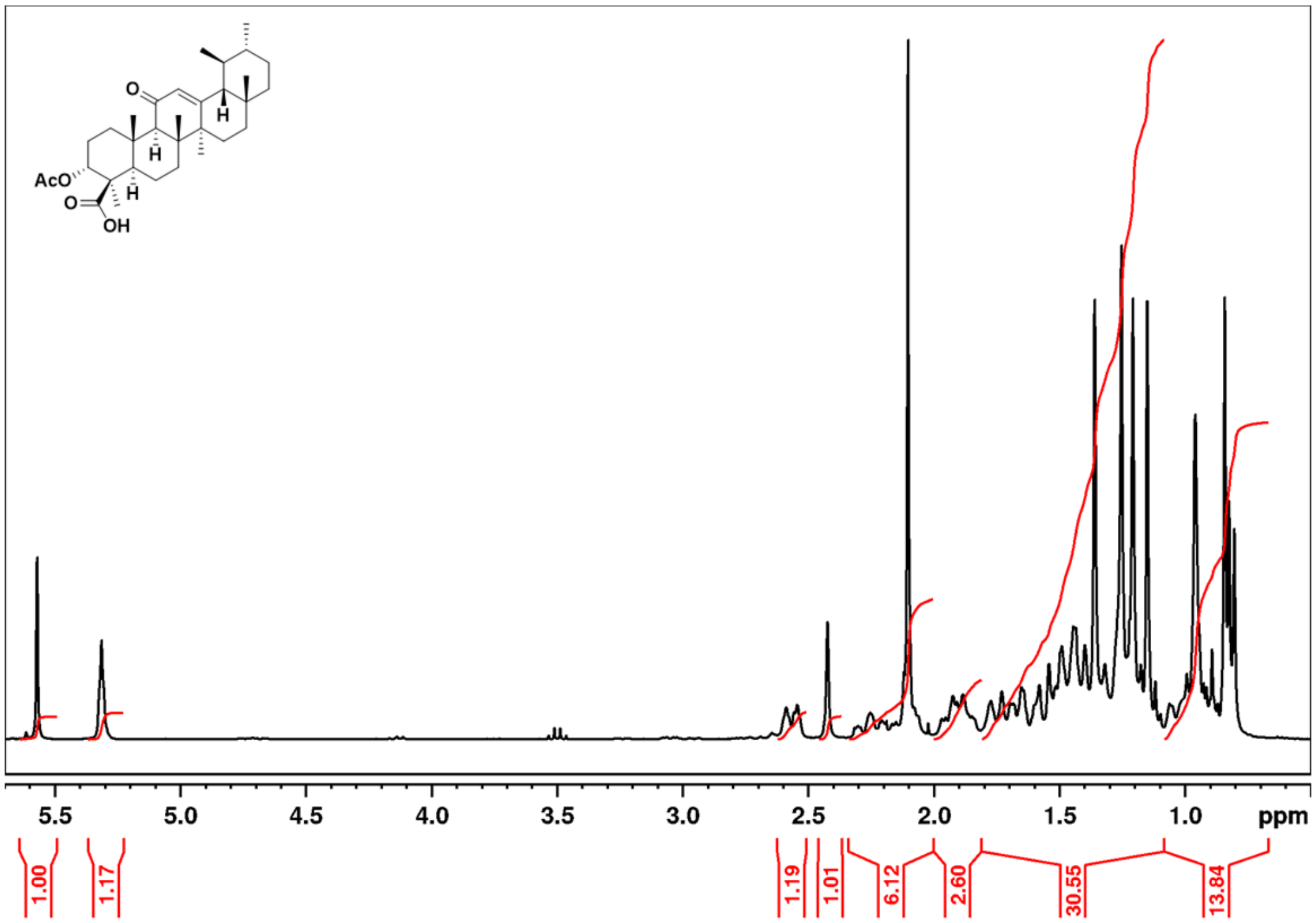

Figure S2: ^{1}H NMR spectrum of AKBA (300 MHz, $CDCl_3$).

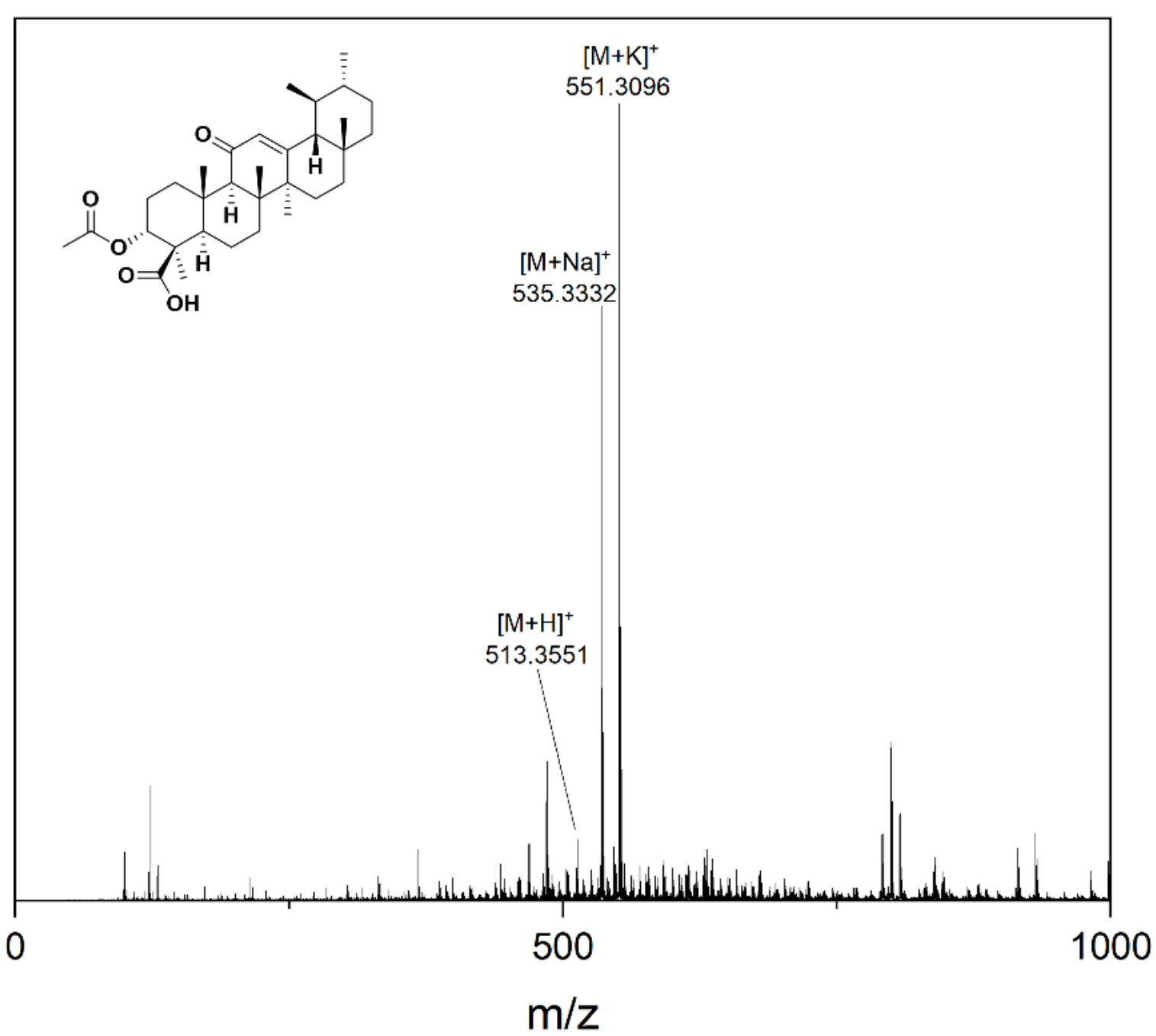

Figure S3: ESI-MS of AKBA.

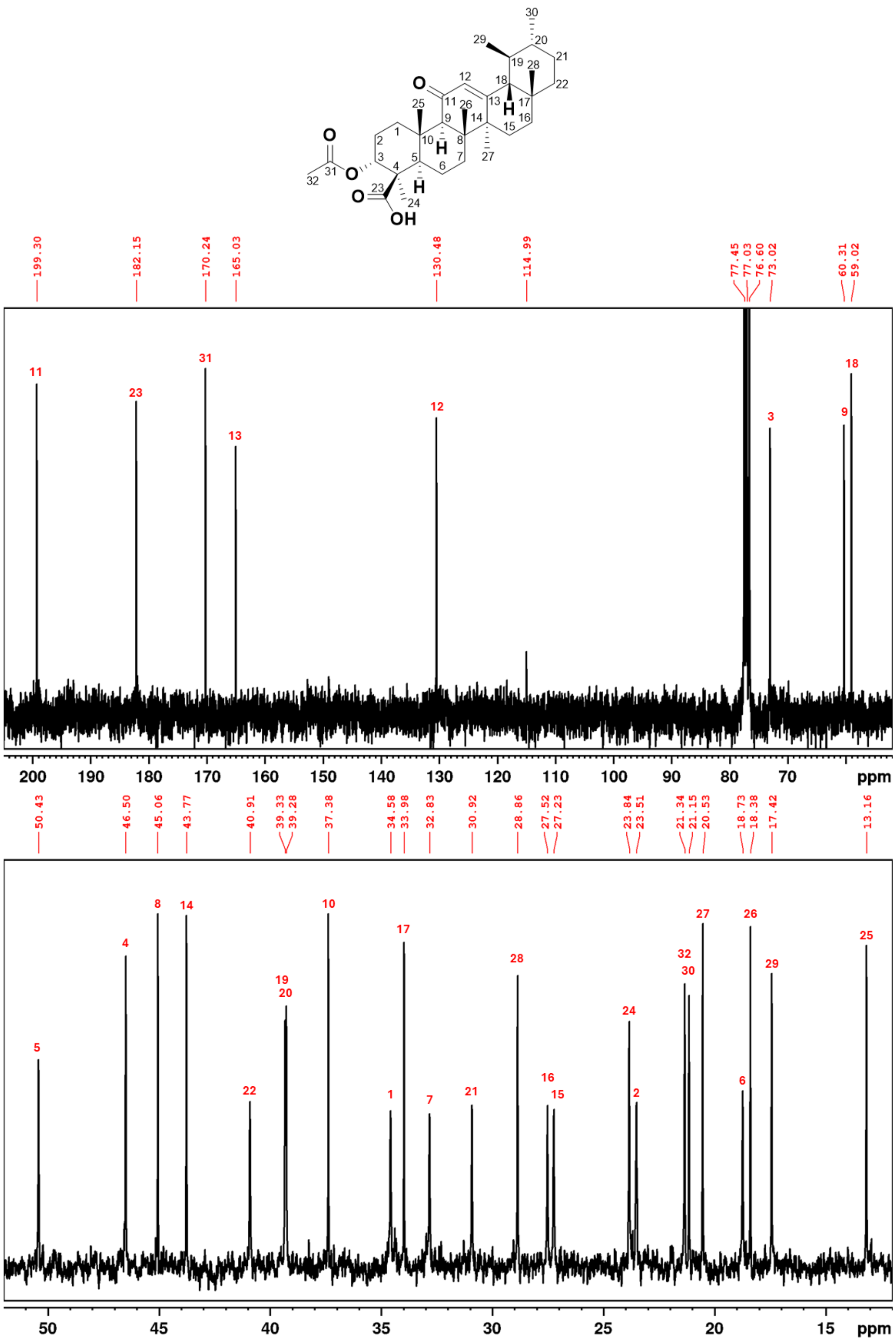

Figure S4: ^{13}C NMR spectrum of AKBA (75 MHz, $CDCl_3$).

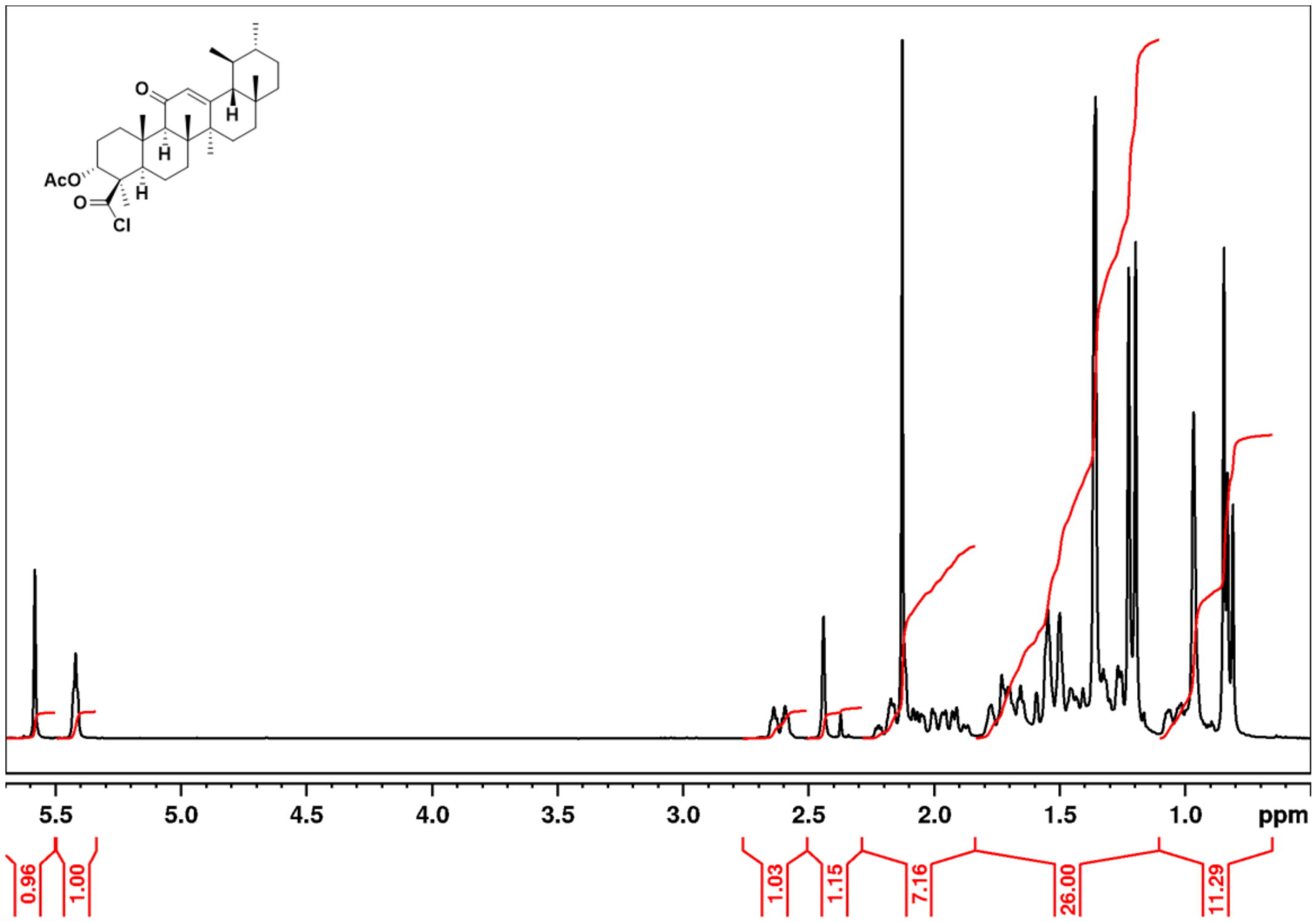

Figure S5: ^{1}H NMR spectrum of AKBA-Cl (300 MHz, $CDCl_3$).

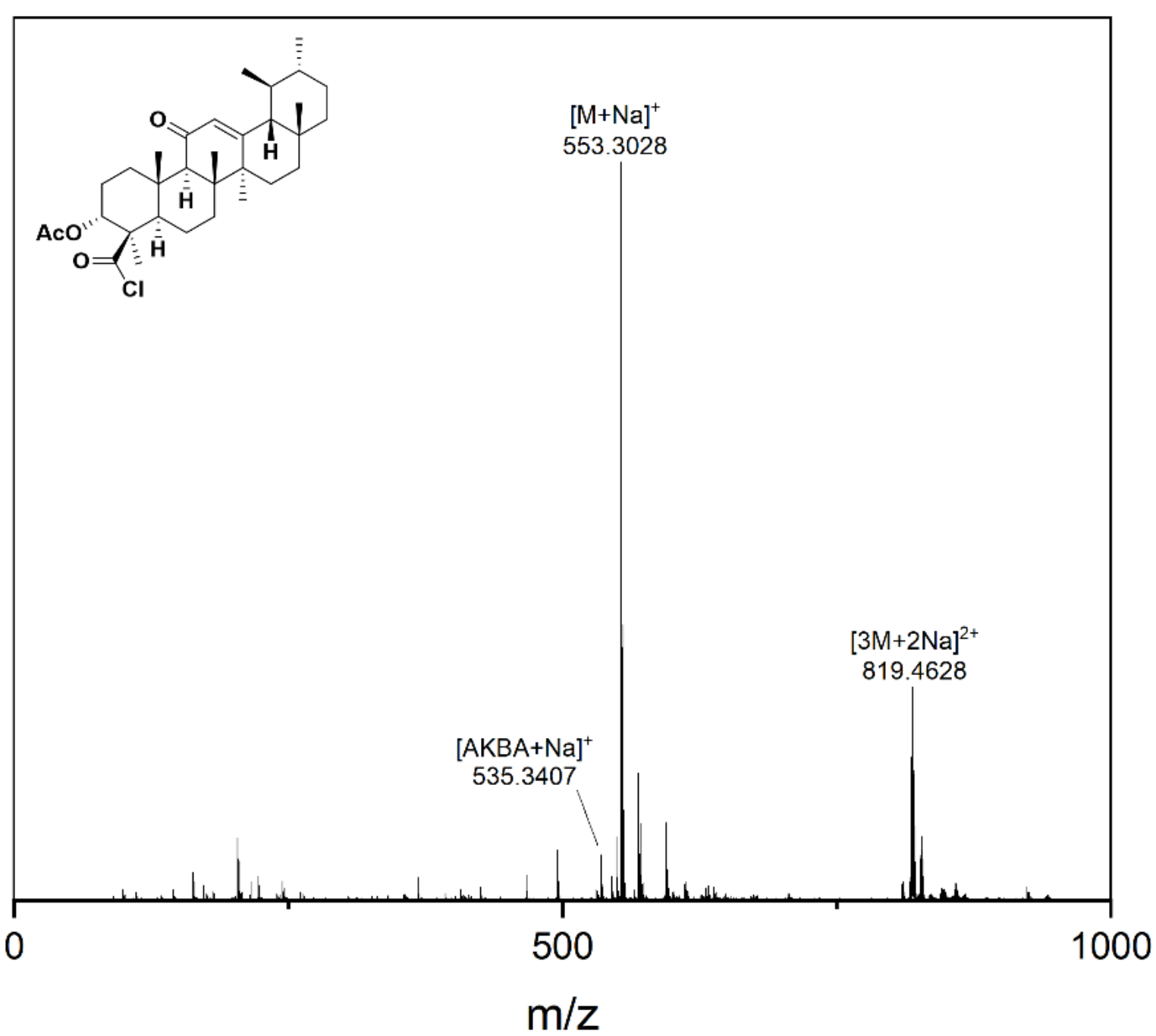

Figure S6: ESI-MS of AKBA-Cl.

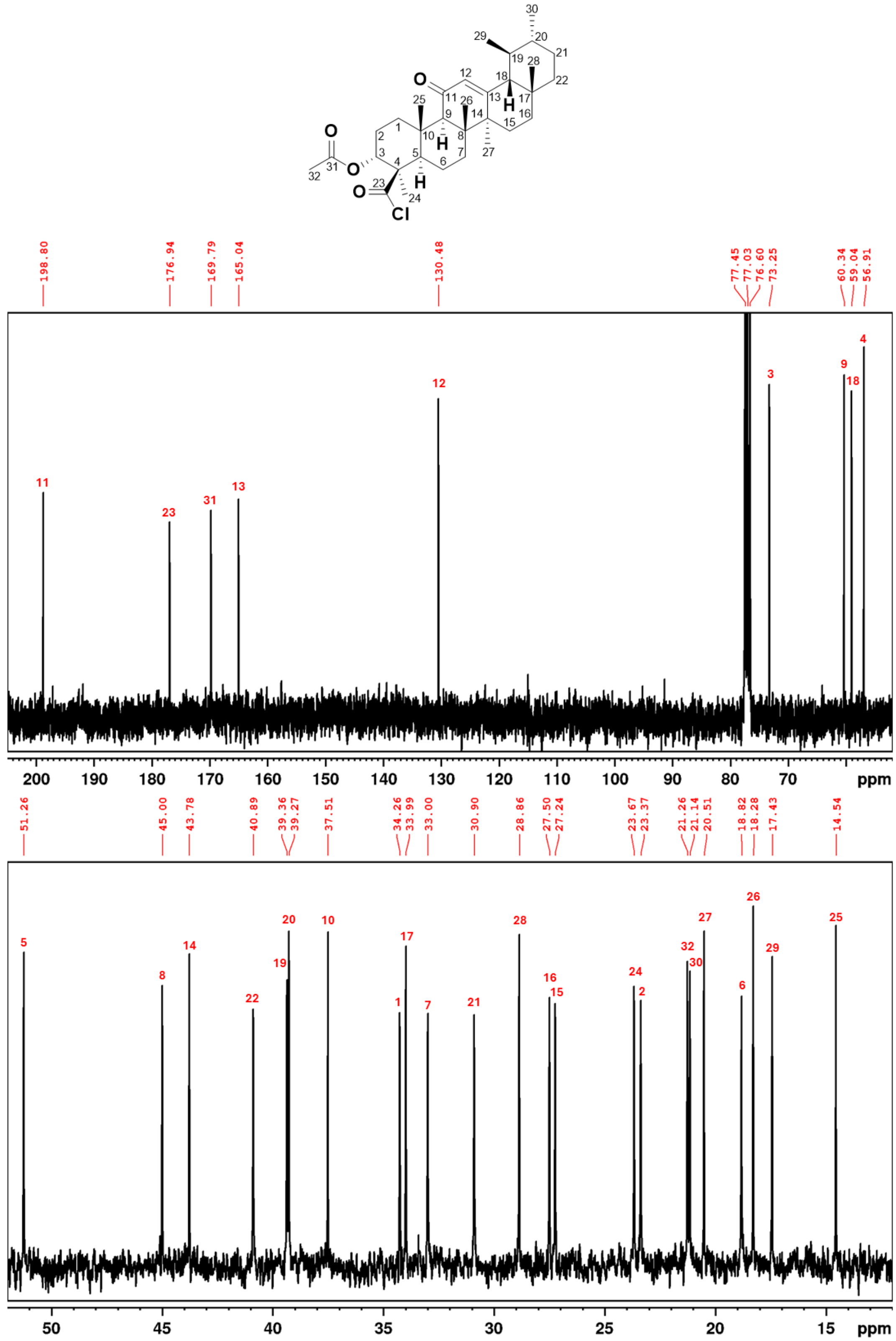

Figure S7: ^{13}C NMR spectrum of AKBA-Cl (75 MHz, $CDCl_3$).

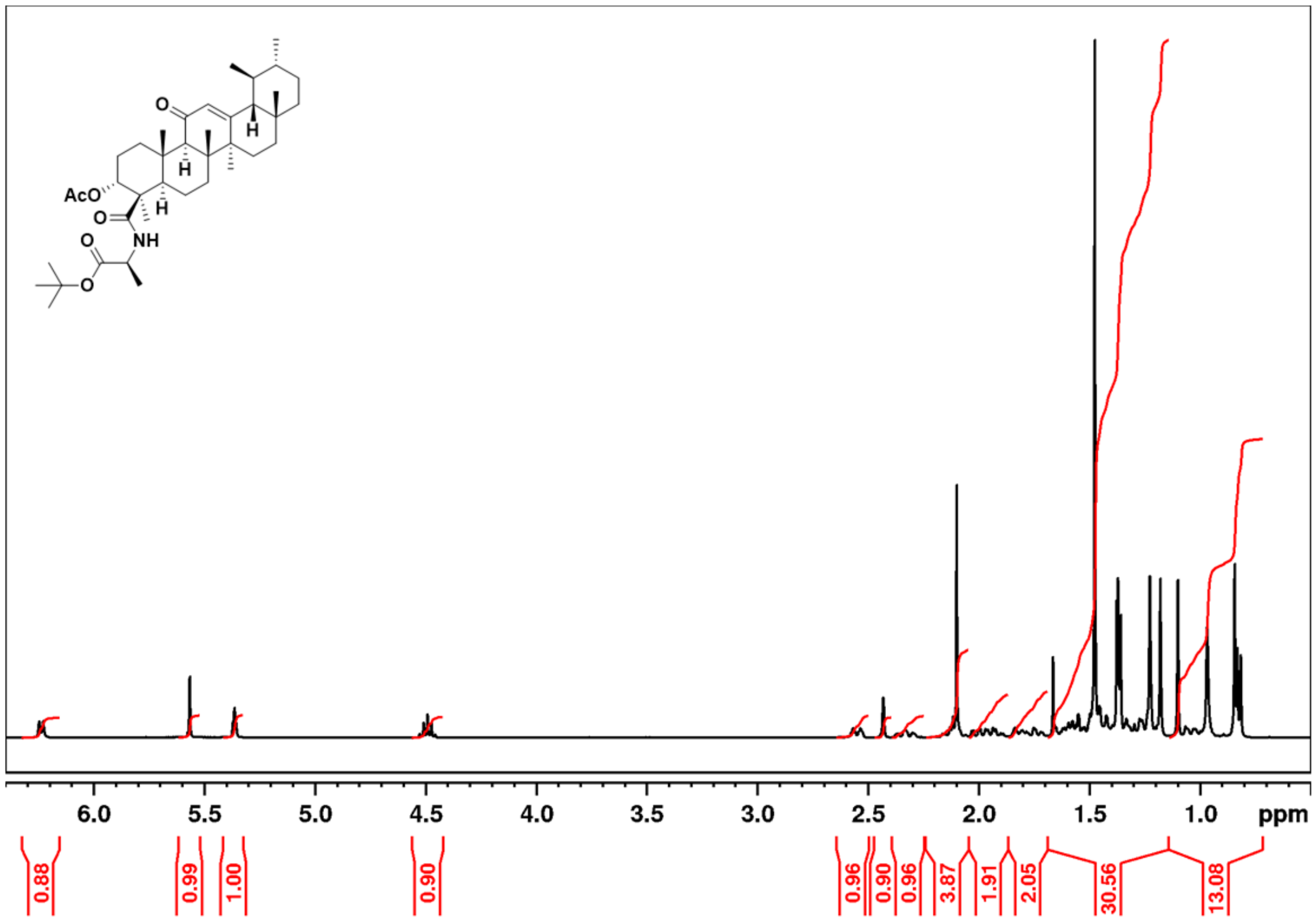

Figure S8: ^{1}H NMR spectrum of AKBA-Ala-*t*-Bu (300 MHz, $CDCl_3$).

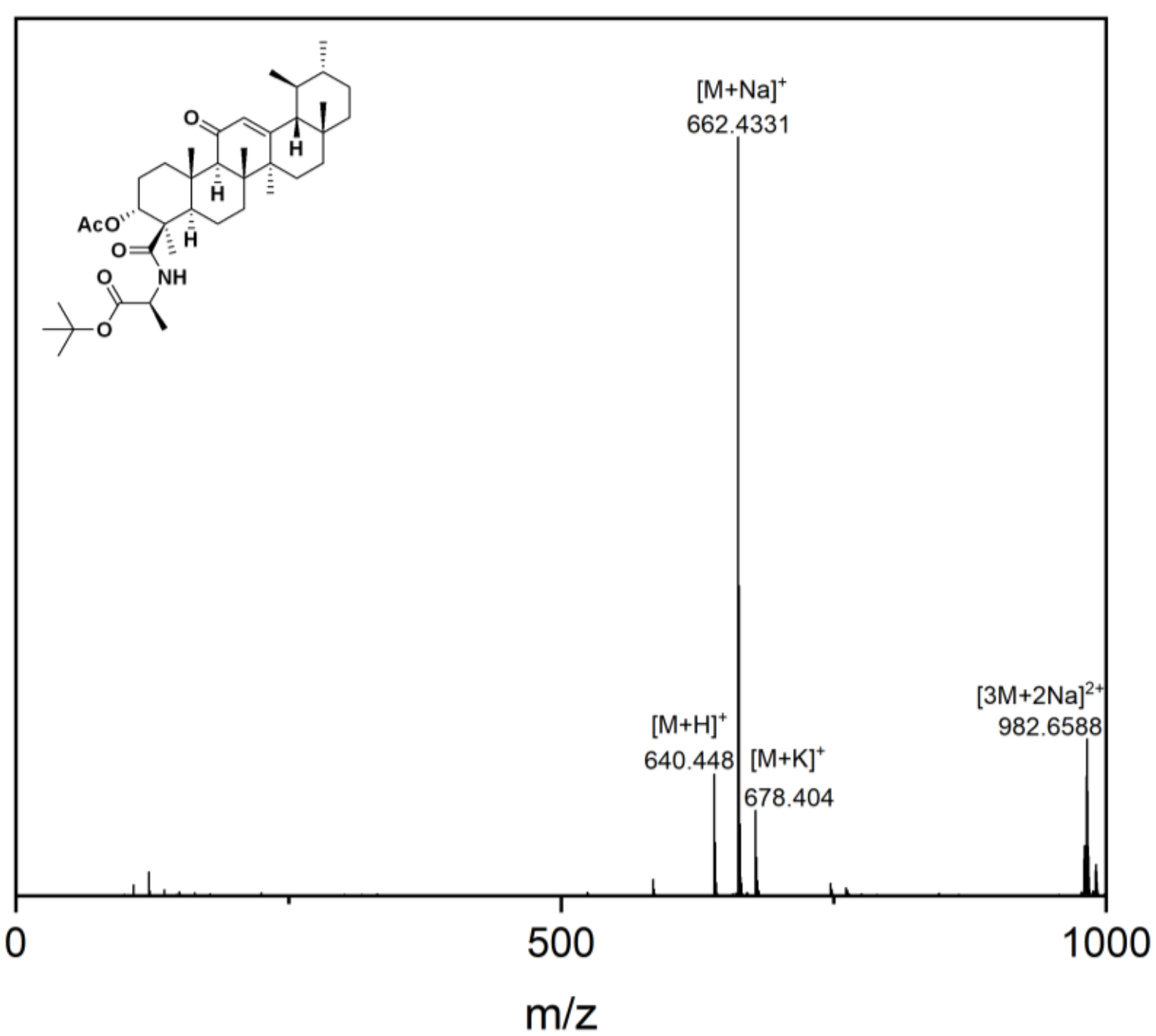

Figure S9: ESI-MS of AKBA-Ala-*t*-Bu.

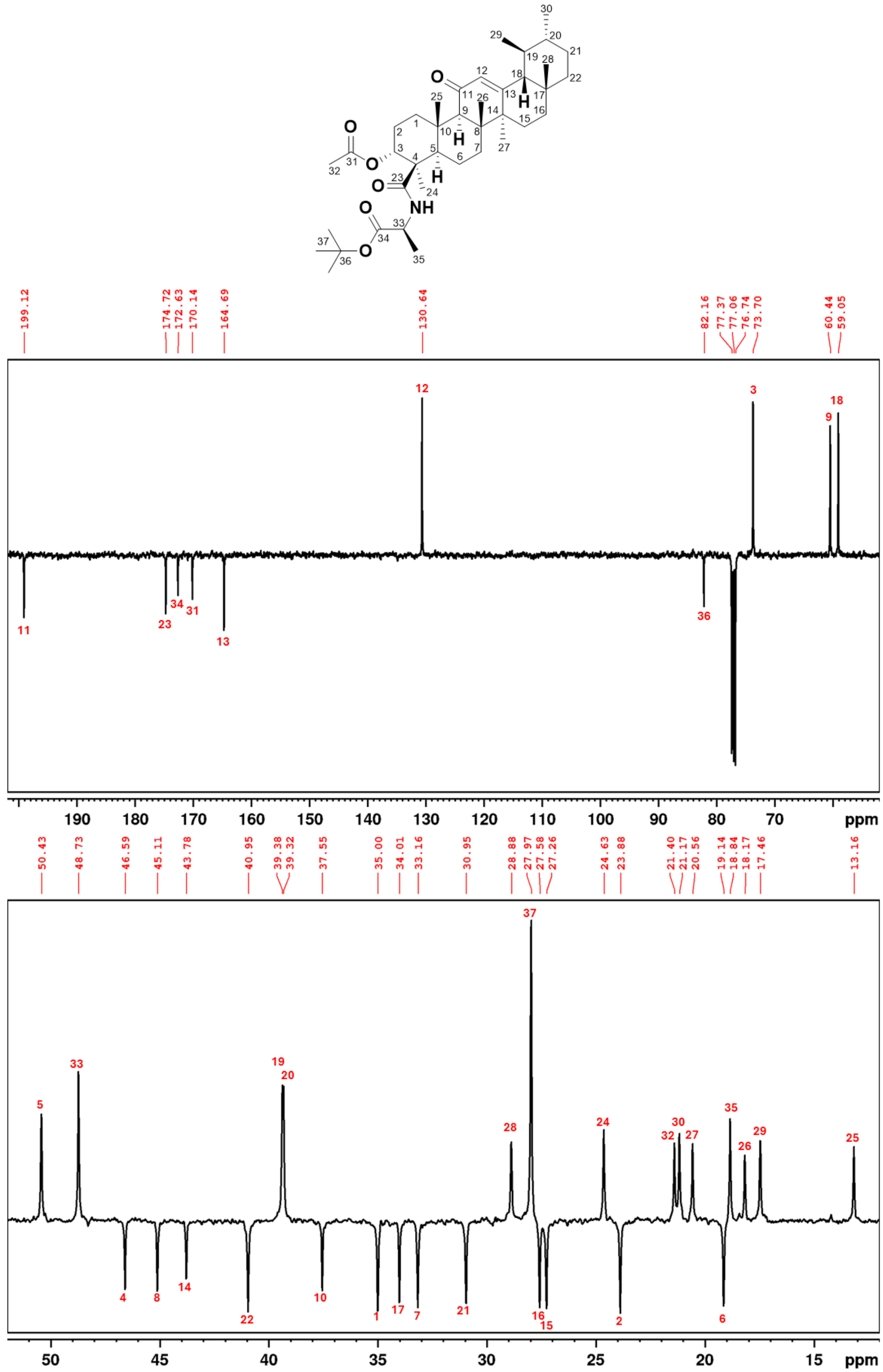

Figure S10: ^{13}C-APT NMR spectrum of AKBA-Ala-*t*-Bu (100 MHz, $CDCl_3$).

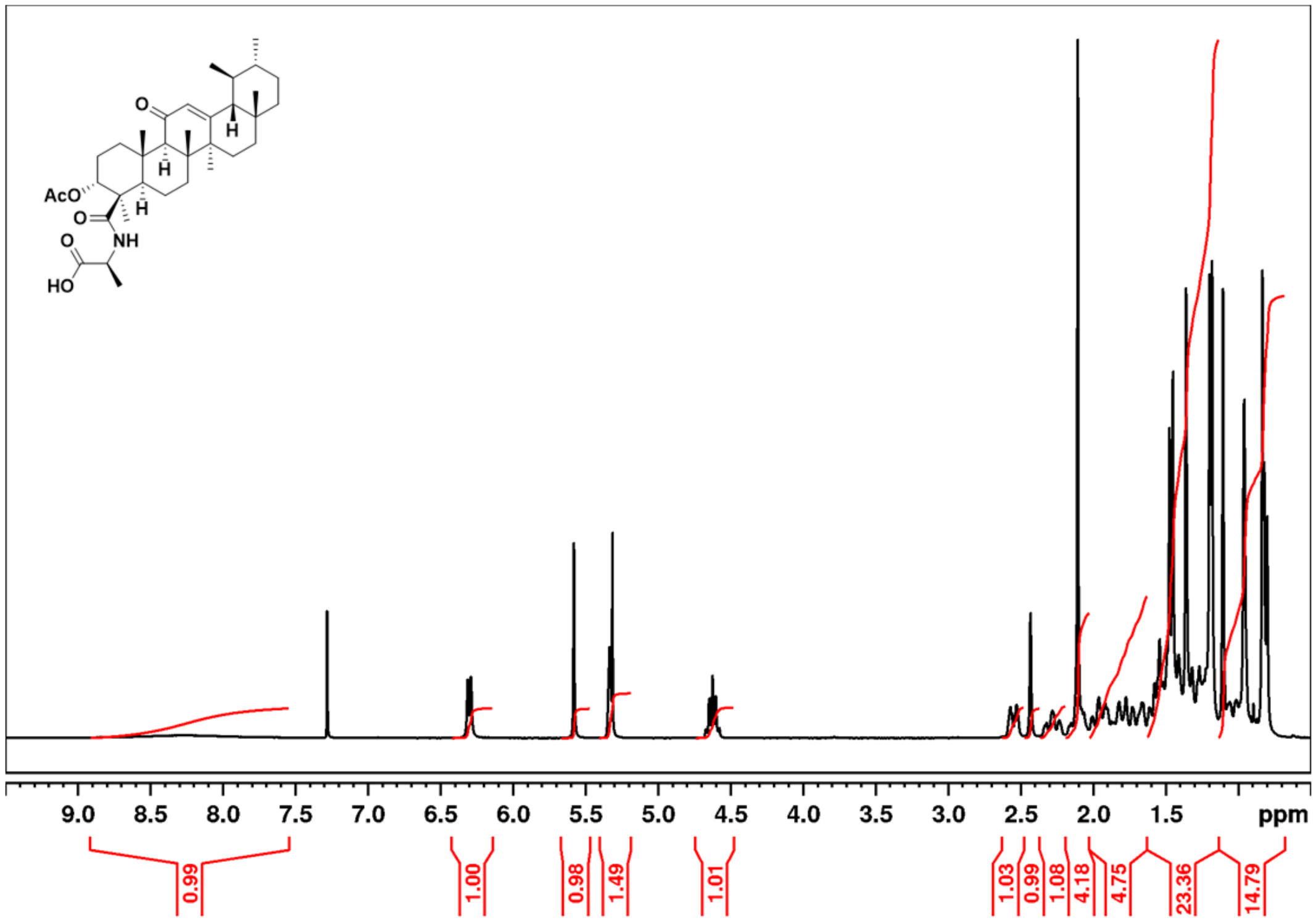

Figure S11: ^{1}H NMR spectrum of AKBA-Ala (400 MHz, $CDCl_3$).

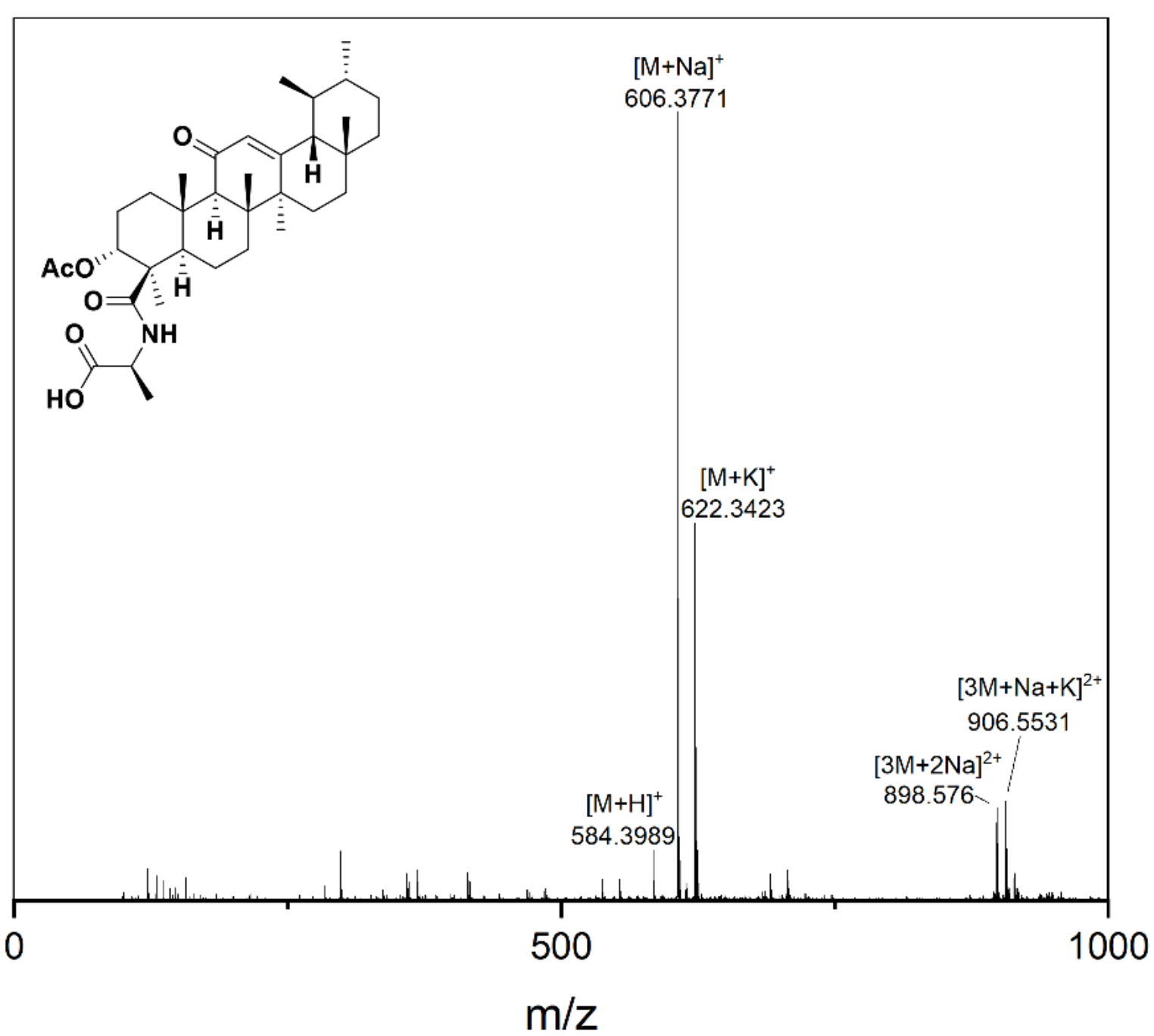

Figure S12: ESI-MS of AKBA-Ala.

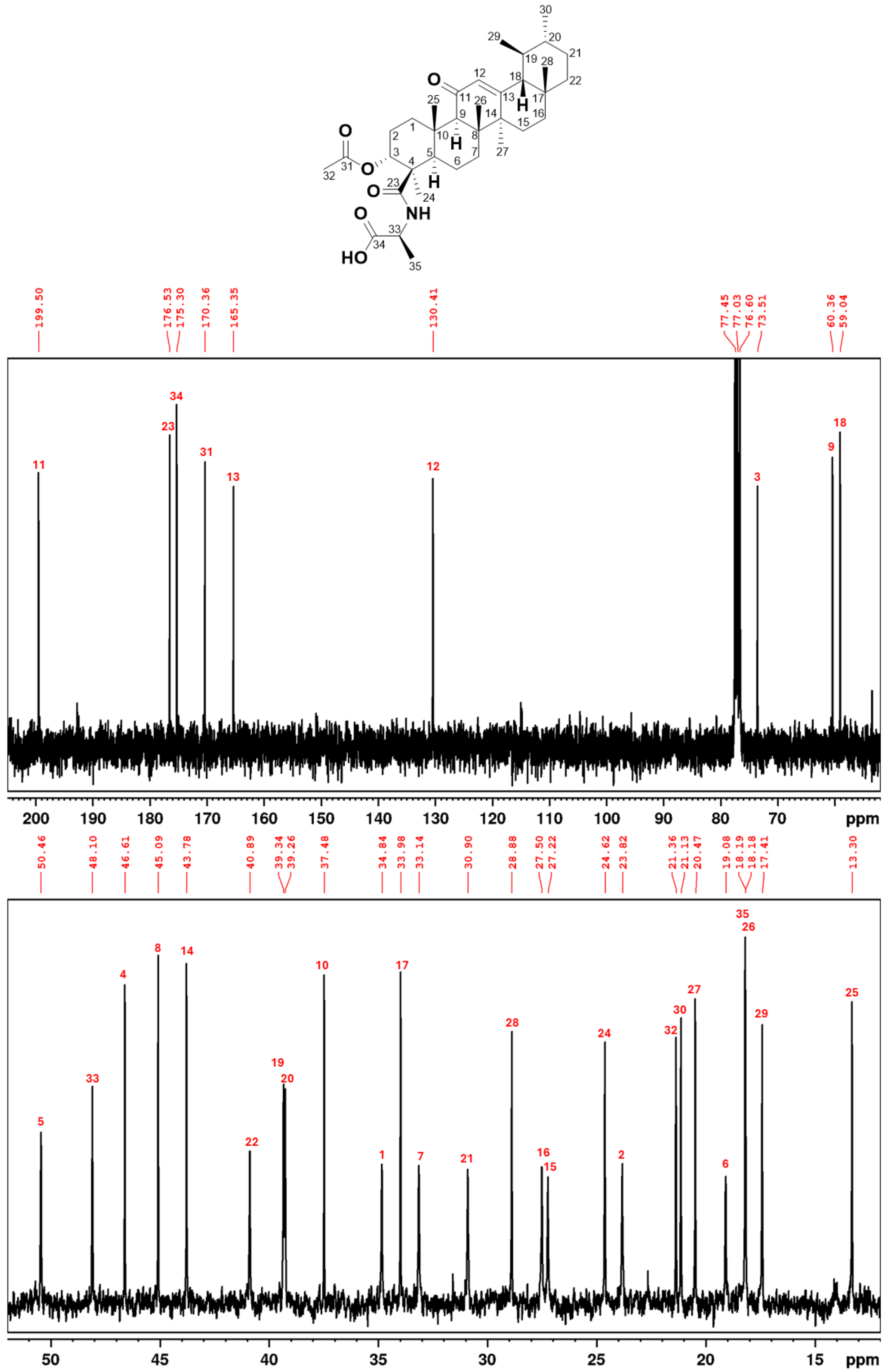

Figure S13: ^{13}C NMR spectrum of AKBA-Ala (100 MHz, $CDCl_3$).

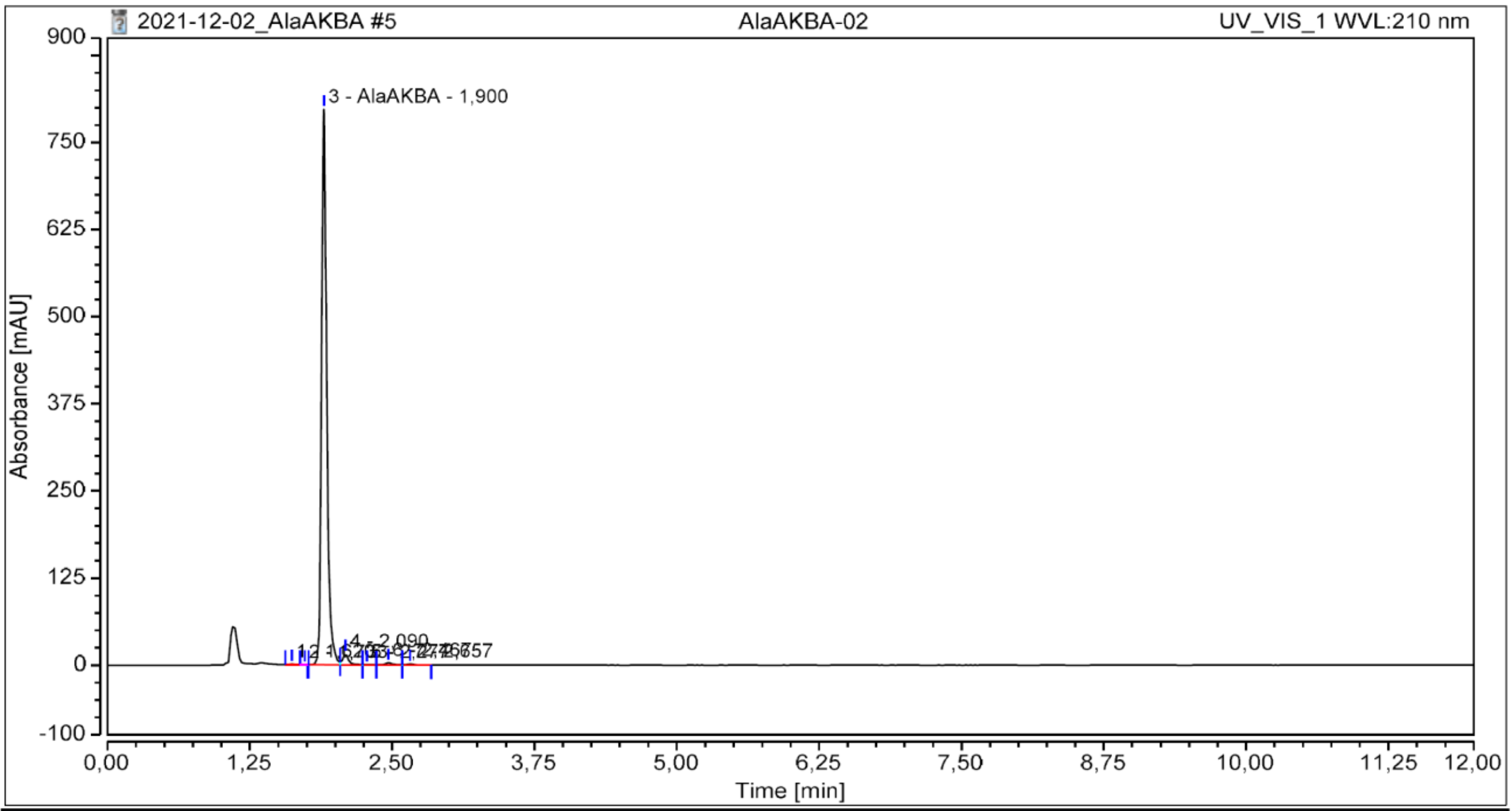

Integration Results

No.	Peak Name	Retention Time<br>min	Area<br>mAU*min	Height<br>mAU	Relative Area<br>%	Relative Height<br>%	Amount
1		1,620	0,057	0,885	0,13	0,11	n.a.
2		1,733	0,003	0,077	0,01	0,01	n.a.
3	AlaAKBA	1,900	41,821	797,282	96,75	97,34	n.a.
4		2,090	0,950	15,626	2,20	1,91	n.a.
5		2,277	0,044	0,522	0,10	0,06	n.a.
6		2,467	0,234	3,146	0,54	0,38	n.a.
7		2,657	0,118	1,564	0,27	0,19	n.a.
**Total:**			**43,226**	**819,101**	**100,00**	**100,00**	

Figure S14: HPLC of AKBA-Ala.

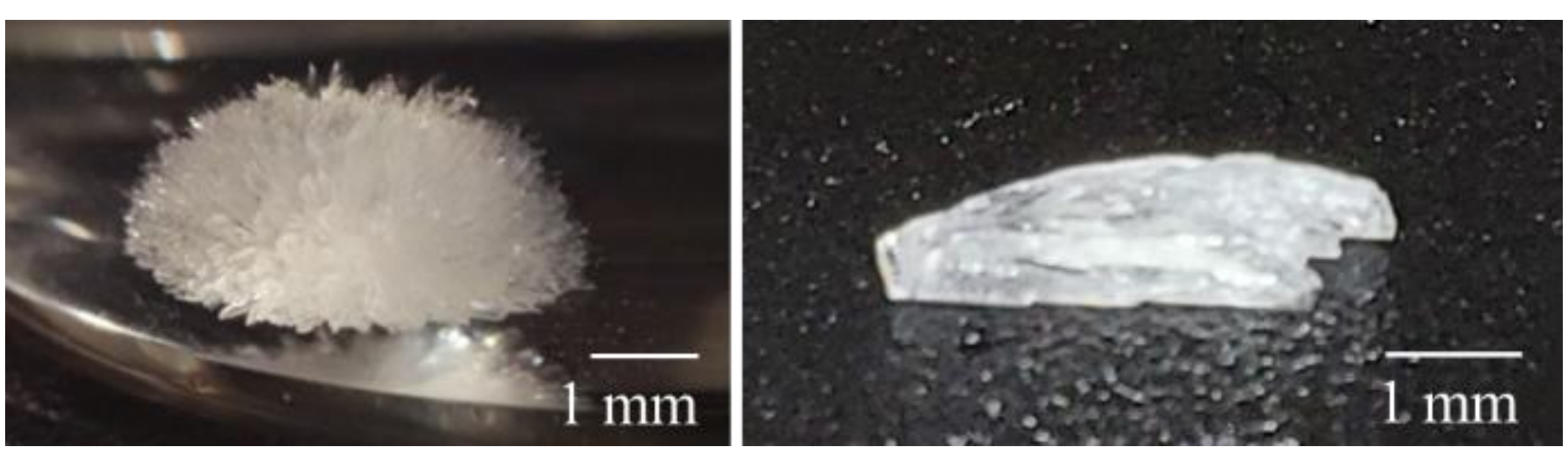

Figure S15: Crystallization of AKBA (left) and a crystal of AKBA-Ala (right).

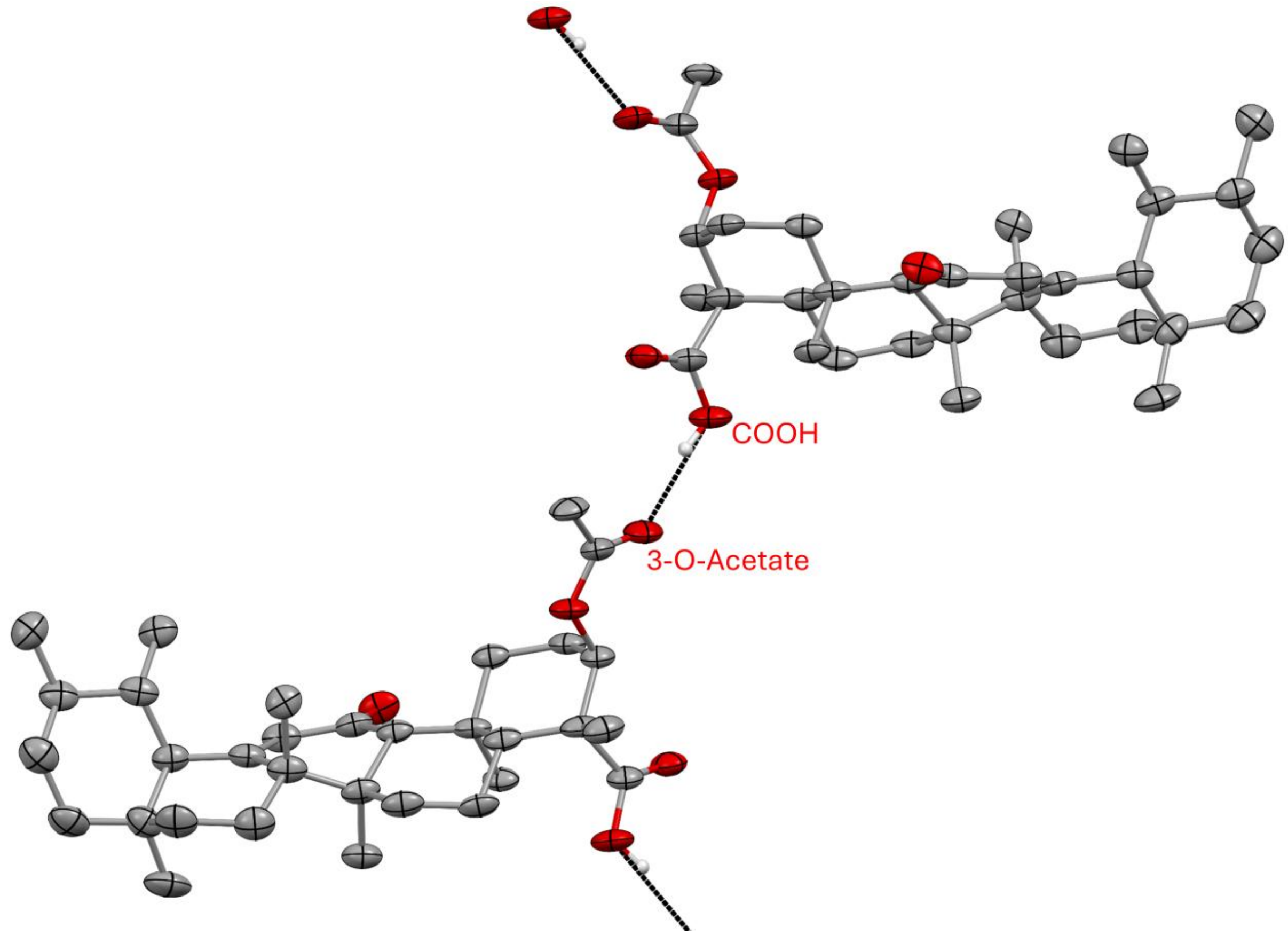

Figure S16: Hydrogen bonding in the solid-state molecular structure of AKBA.

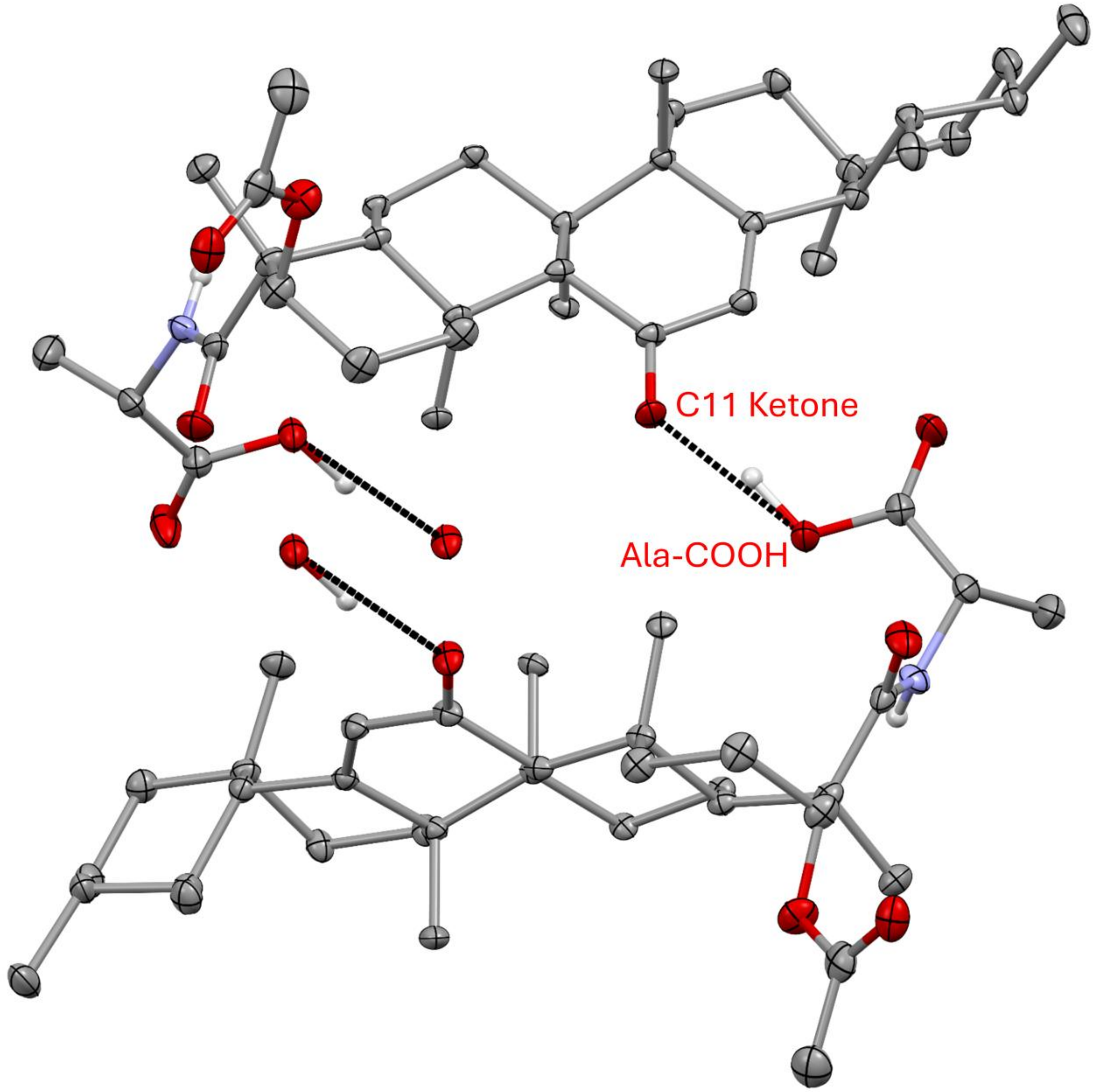

Figure S17: Hydrogen bonding in the solid-state molecular structure of AKBA-Ala.

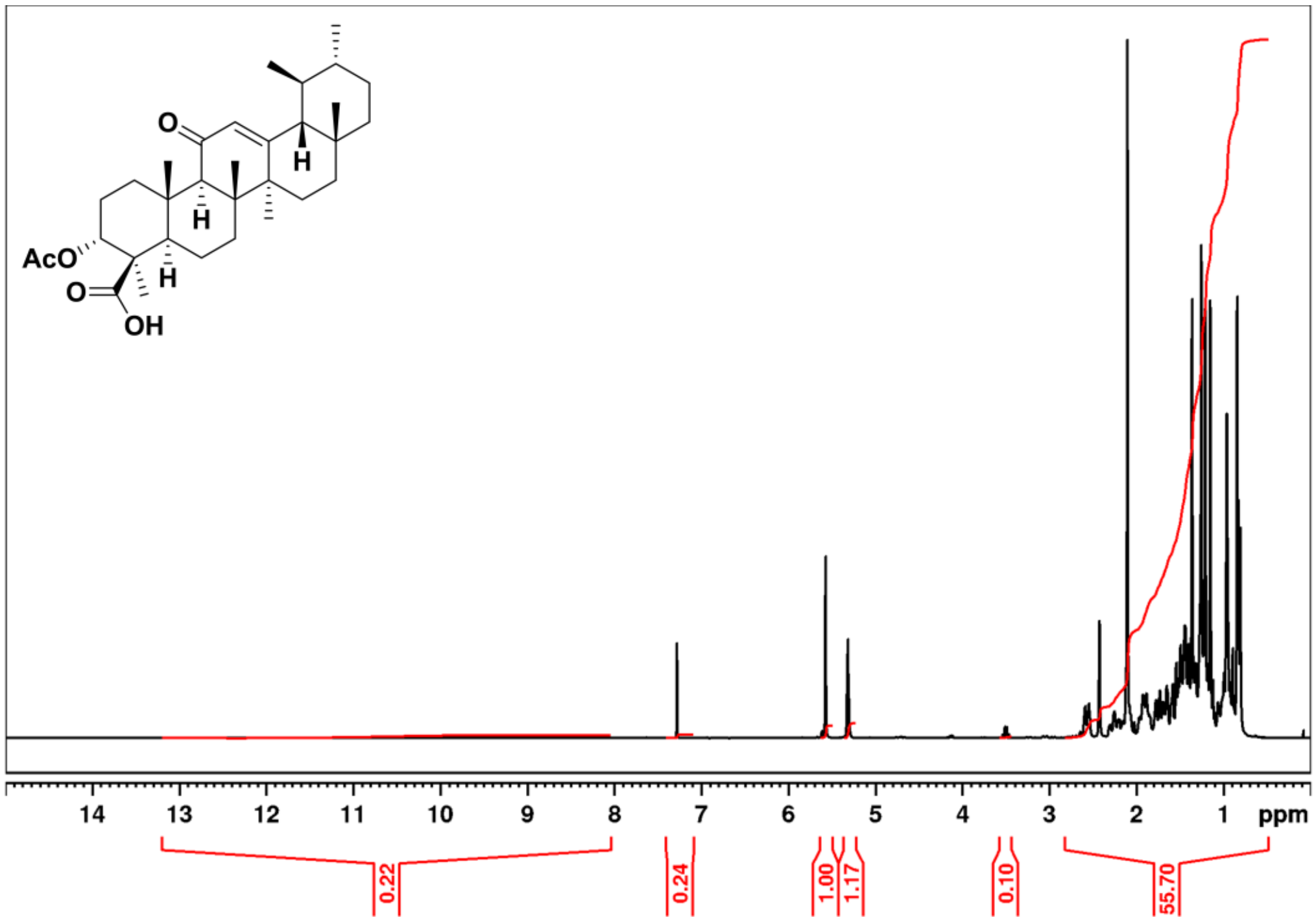

Figure S18: Full ^{1}H NMR spectrum of AKBA (300 MHz, $CDCl_3$).

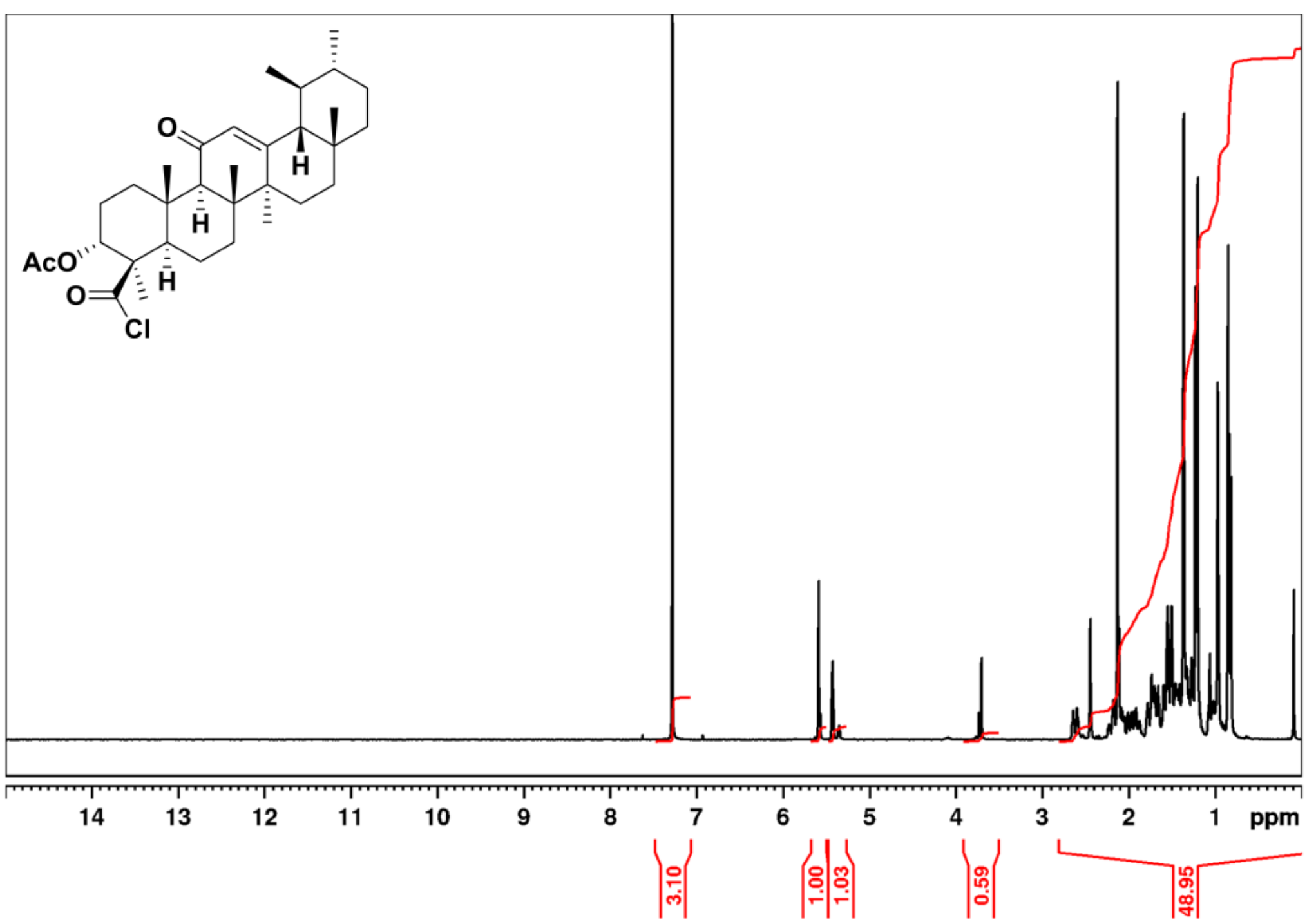

Figure S19: Full ^{1}H NMR spectrum of AKBA-Cl (300 MHz, $CDCl_3$).

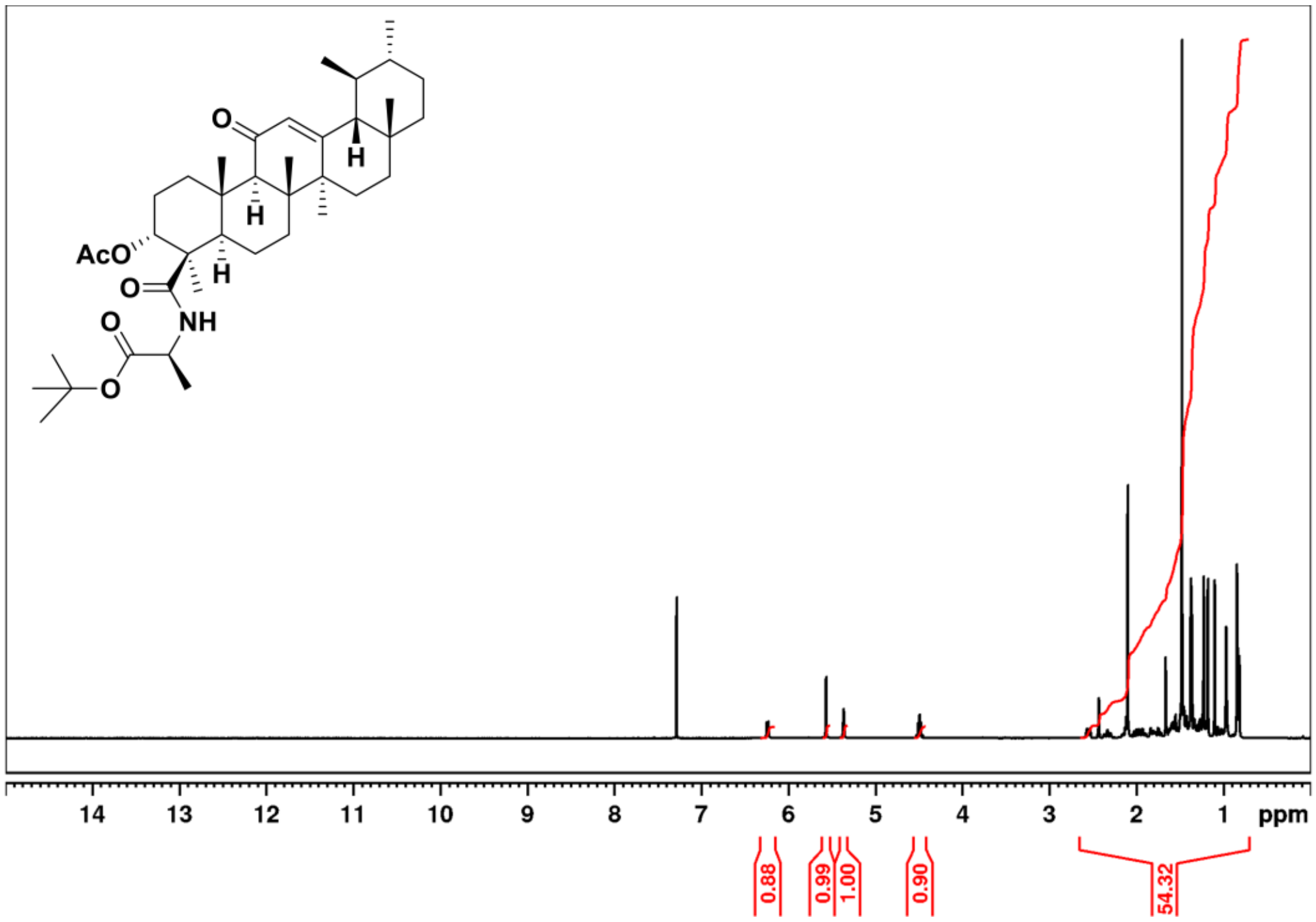

Figure S20: Full ^{1}H NMR spectrum of AKBA-Ala-*t*-Bu (300 MHz, $CDCl_3$).

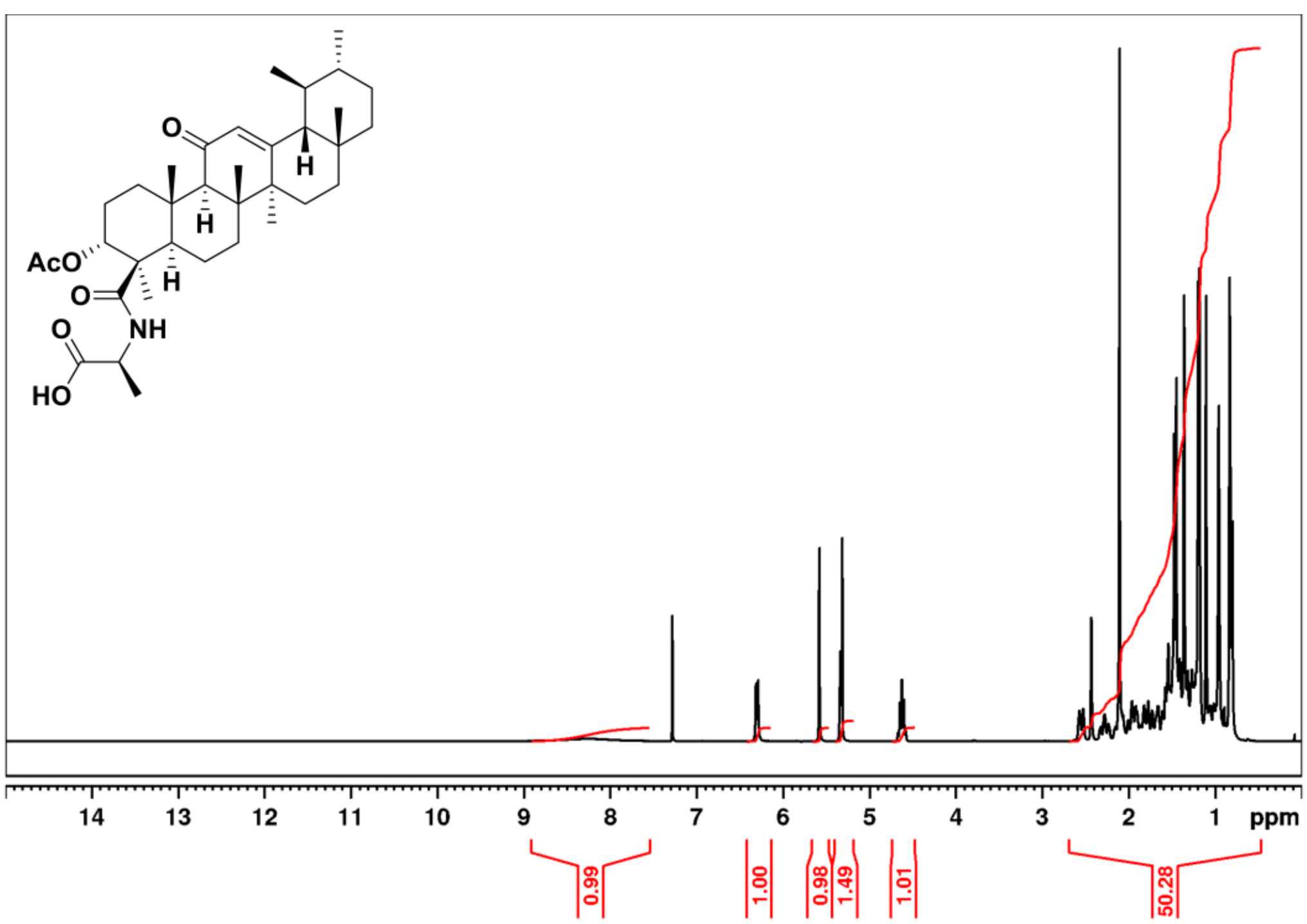

Figure S21: Full ^{1}H NMR spectrum of AKBA-Ala (400 MHz, $CDCl_3$).

References

1. TLC stain recipes by Vanderbilt university. https://www.vanderbilt.edu/AnS/Chemistry/Rizzo/stuff/TLC.html (accessed 08.05.2026).
2. Hooft, R.; Nonius, B. *Collect: Data collection software*, BV Nonius: The Netherlands, **1998**.
3. Otwinowski, Z.; Minor, W. Processing of X-ray diffraction data collected in oscillation mode. In *Methods Enzymol.*; Elsevier: **1997**; Vol. 276, pp 307-326.
4. *APEX4 and SADABS*, Bruker AXS Inc.: Madison, Wisconsin, USA, **2001**.
5. Krause, L.; Herbst-Irmer, R.; Sheldrick, G. M.; Stalke, D. *J. Appl. Crystallogr.* **2015**, *48*, 3-10.
6. Sheldrick, G. M. *Acta Crystallogr. A* **2015**, *71*, 3-8.3.
7. Sheldrick, G. M. *Acta Crystallogr. C* **2015**, *71*, 3-8.
8. Dolomanov, O. V.; Bourhis, L. J.; Gildea, R. J.; Howard, J. A.; Puschmann, H. *J. Appl. Crystallogr.* **2009**, *42*, 339-341.
9. Jauch, J.; Bergmann, J. *Eur. J. Org. Chem.* **2003**, *2003*, 4752-4756.
10. Belsner, K.; Büchele, B.; Werz, U.; Syrovets, T.; Simmet, T. *Magn. Reson. Chem.* **2003**, *41*, 115-122.
11. Günther, K.; Ehrhardt, C.; Werz, O.; Jordan, P. M. *STAR Protoc* **2024**, *5*, 103142.
12. Werz, O.; Bürkert, E.; Samuelsson, B.; Rådmark, O.; Steinhilber, D. *Blood* **2002**, *99*, 1044-1052.
13. Werner, M.; Jordan, P. M.; Romp, E.; Czapka, A.; Rao, Z.; Kretzer, C.; Koeberle, A.; Garscha, U.; Pace, S.; Claesson, H.-E. *FASEB J.* **2019**, *33*, 6140.
14. Parsons, S.; Flack, H. D.; Wagner, T. *Struct. Sci.* **2013**, *69*, 249-259.
